# Supplementary figures and images for: The rubber tree kinome: Genome-wide characterization and insights into coexpression patterns associated with abiotic stress responses
Source: Front Plant Sci. 2023 Feb 7;14:1068202. doi: 10.3389/fpls.2023.1068202 (PMC9941580; doi:10.3389/fpls.2023.1068202)

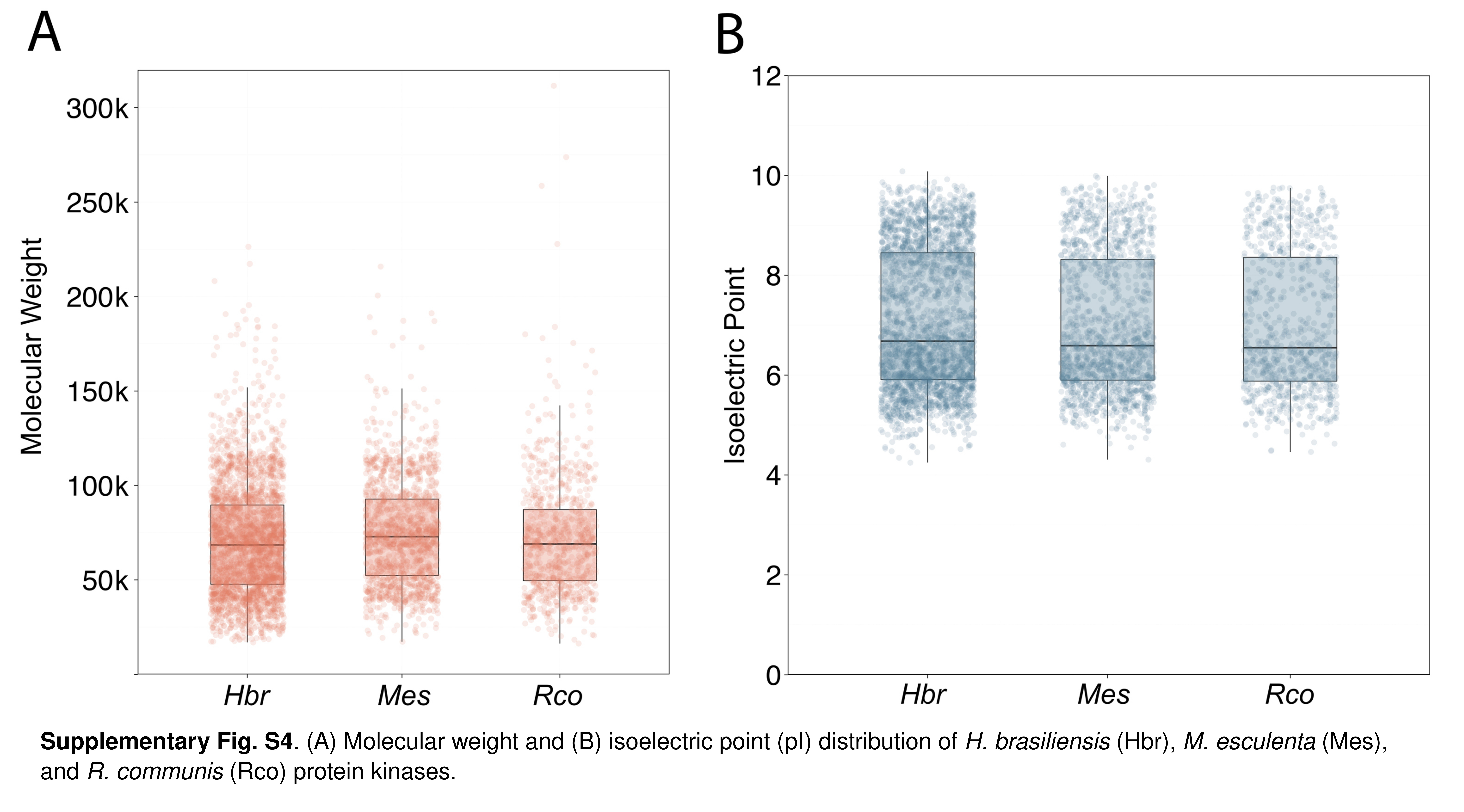

Supplement: Supplementary file 4 [file Image_4.jpeg]

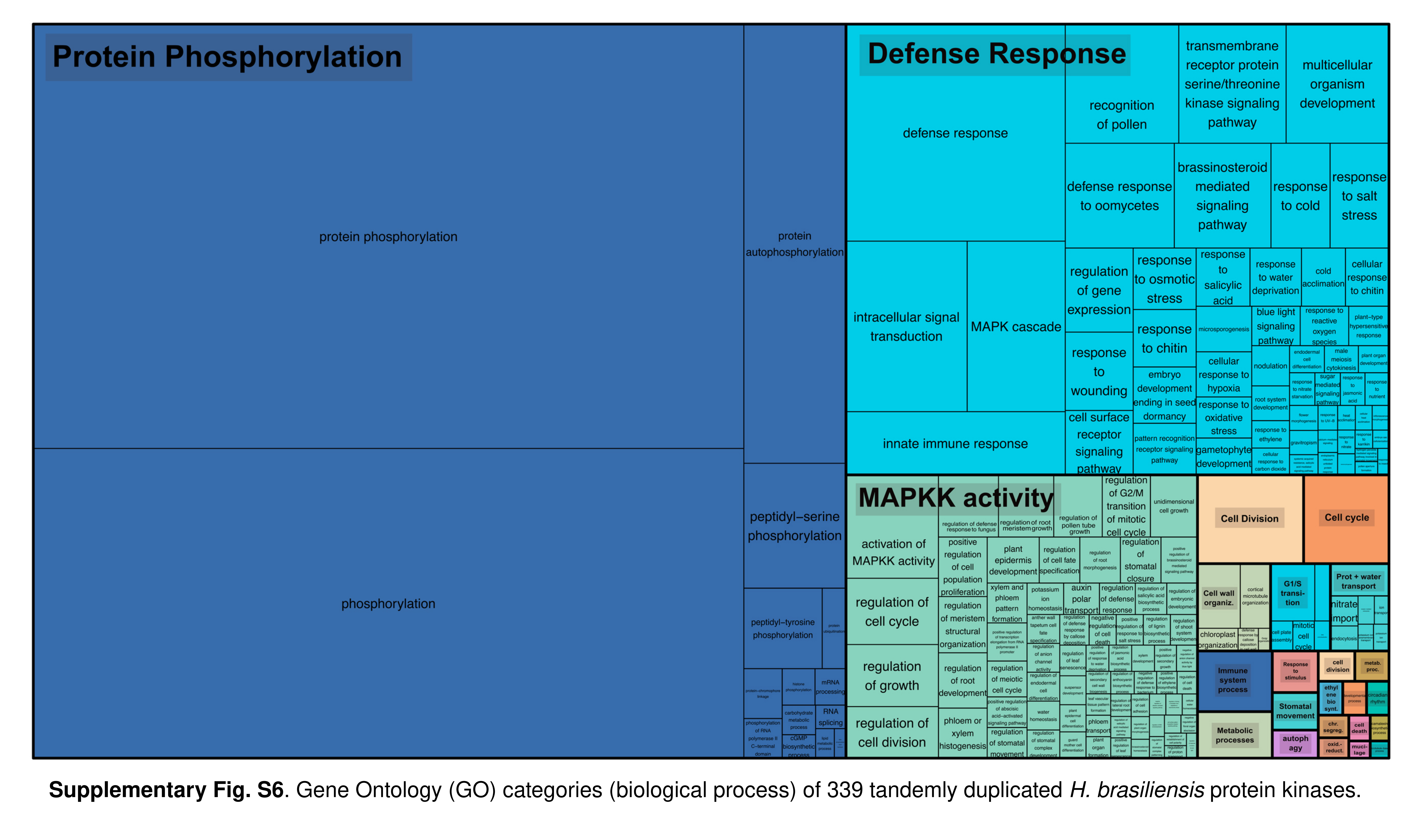

Supplement: Supplementary file 6 [file Image_6.jpeg]

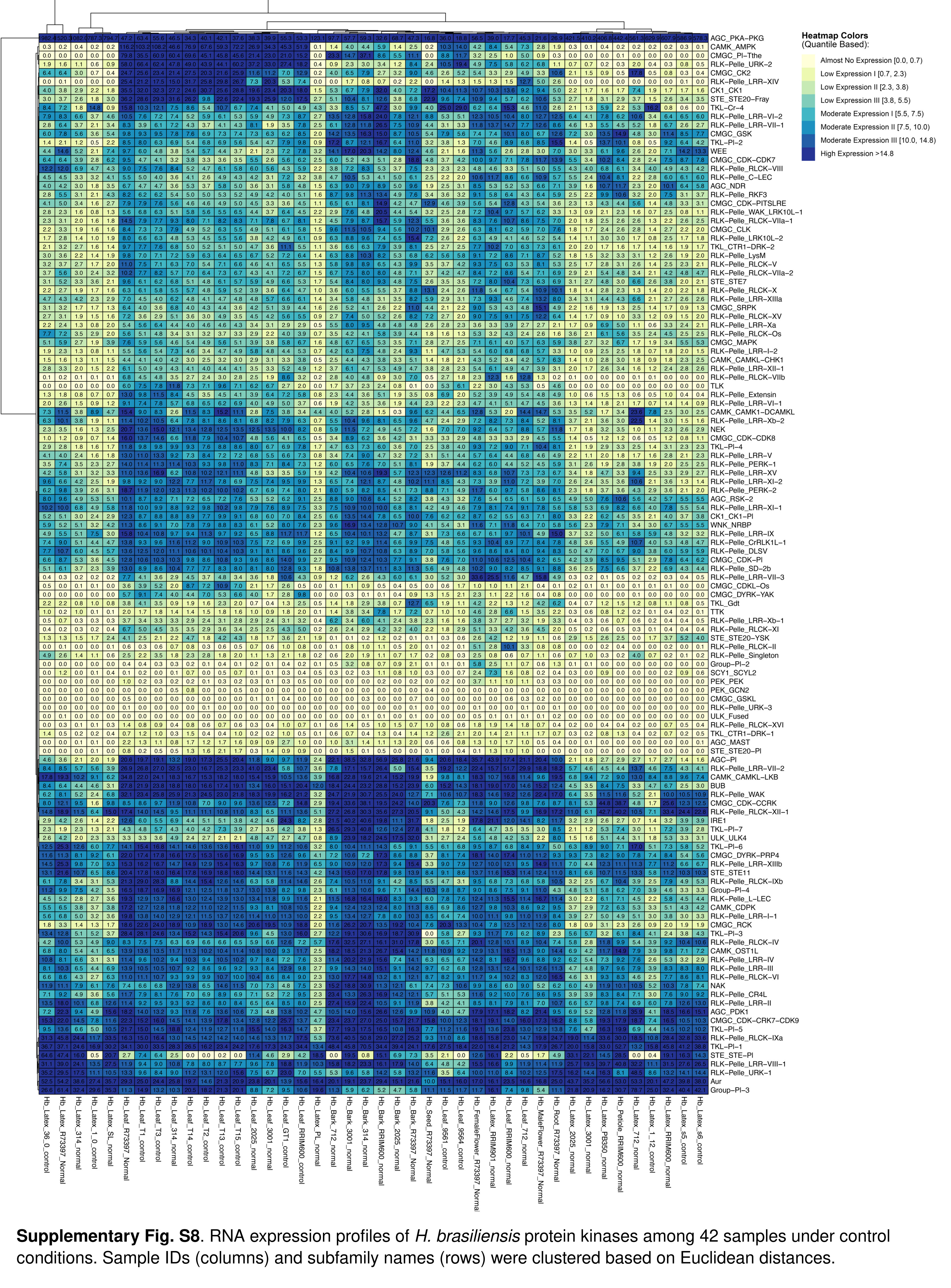

Supplement: Supplementary file 8 [file Image_8.jpeg]

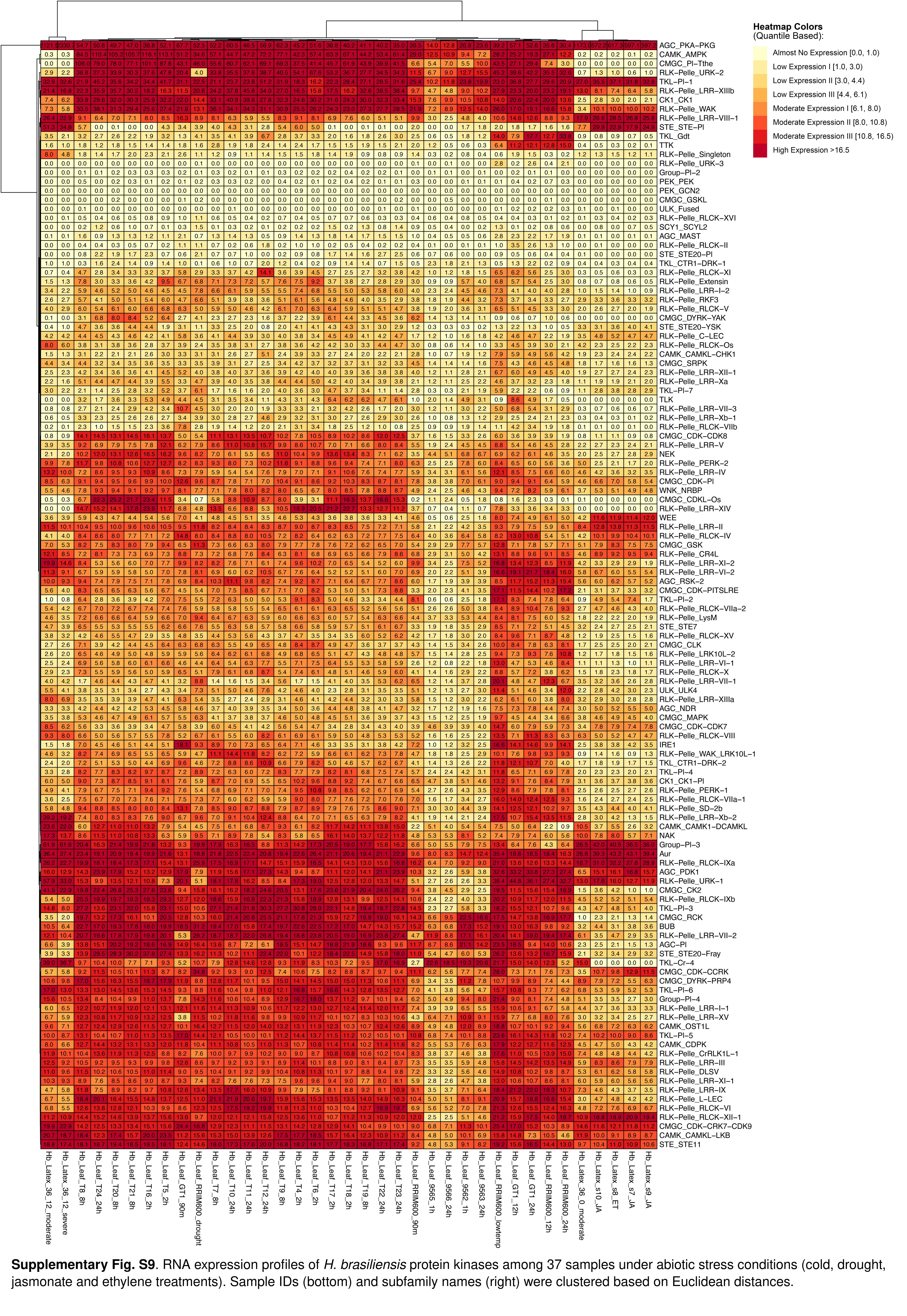

Supplement: Supplementary file 9 [file Image_9.jpeg]

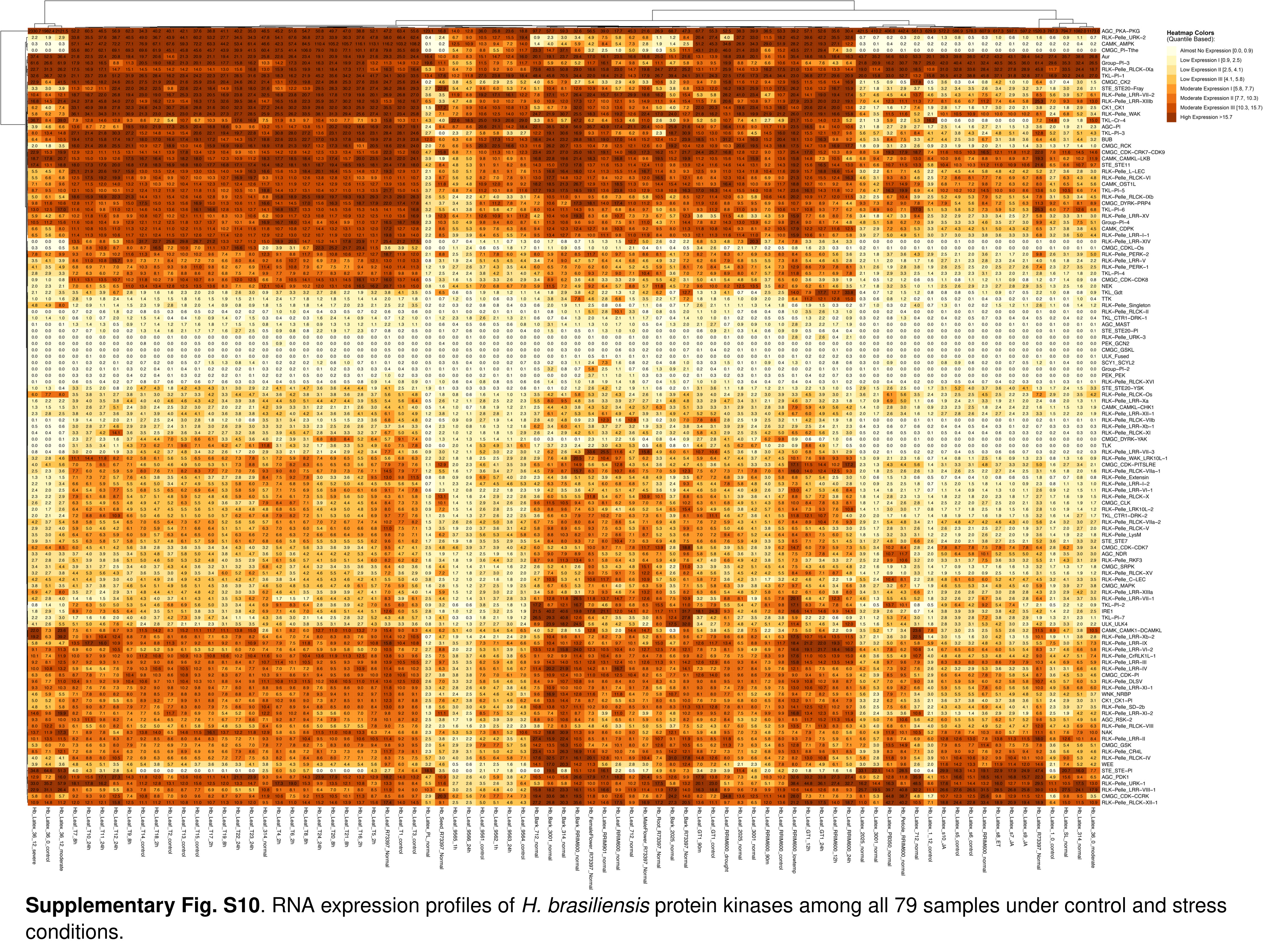

Supplement: Supplementary file 10 [file Image_10.jpeg]
