# Supplementary material for: The rubber tree kinome: Genome-wide characterization and insights into coexpression patterns associated with abiotic stress responses
Source: Front Plant Sci. 2023 Feb 7;14:1068202. doi: 10.3389/fpls.2023.1068202 (PMC9941580; doi:10.3389/fpls.2023.1068202)
Supplement: Supplementary file 1 [file Image_1.pdf]

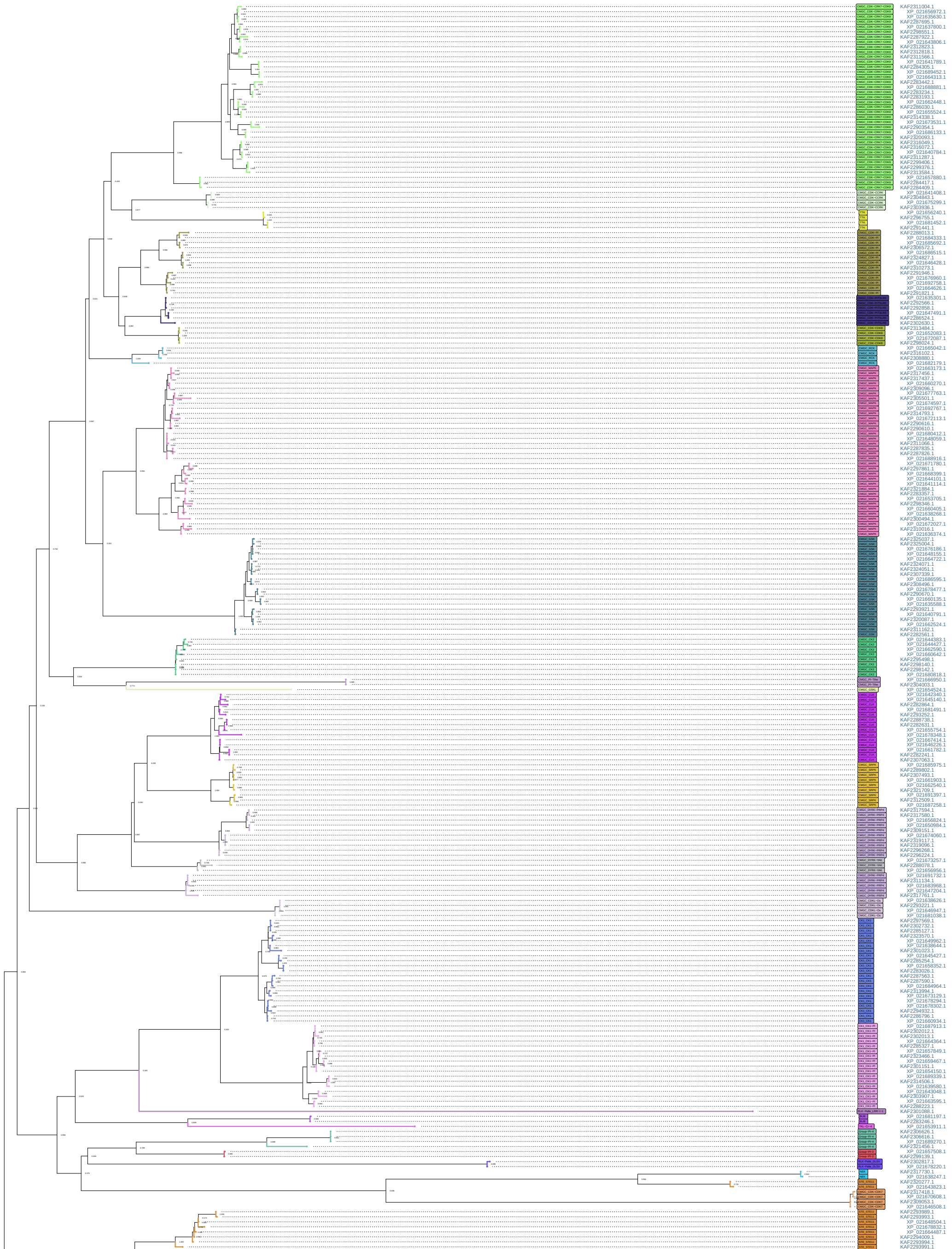

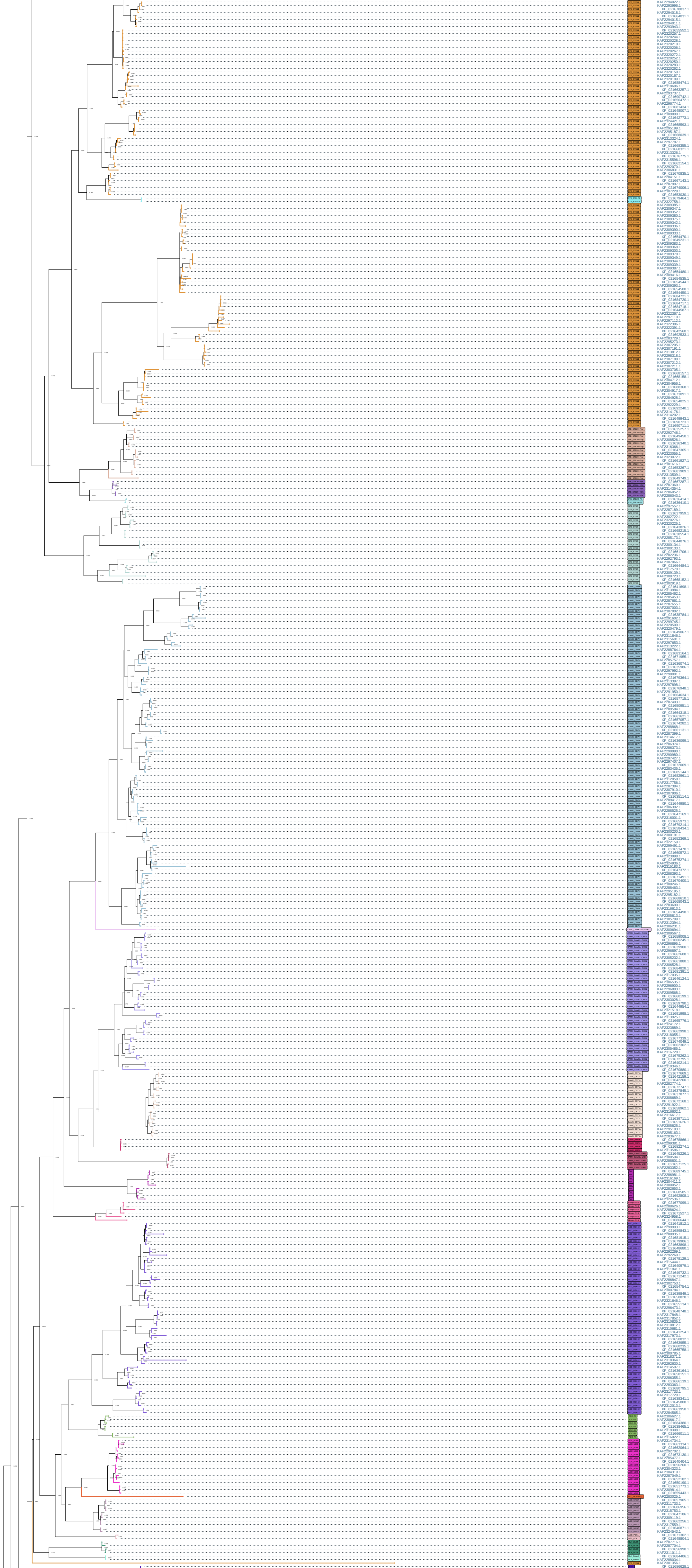

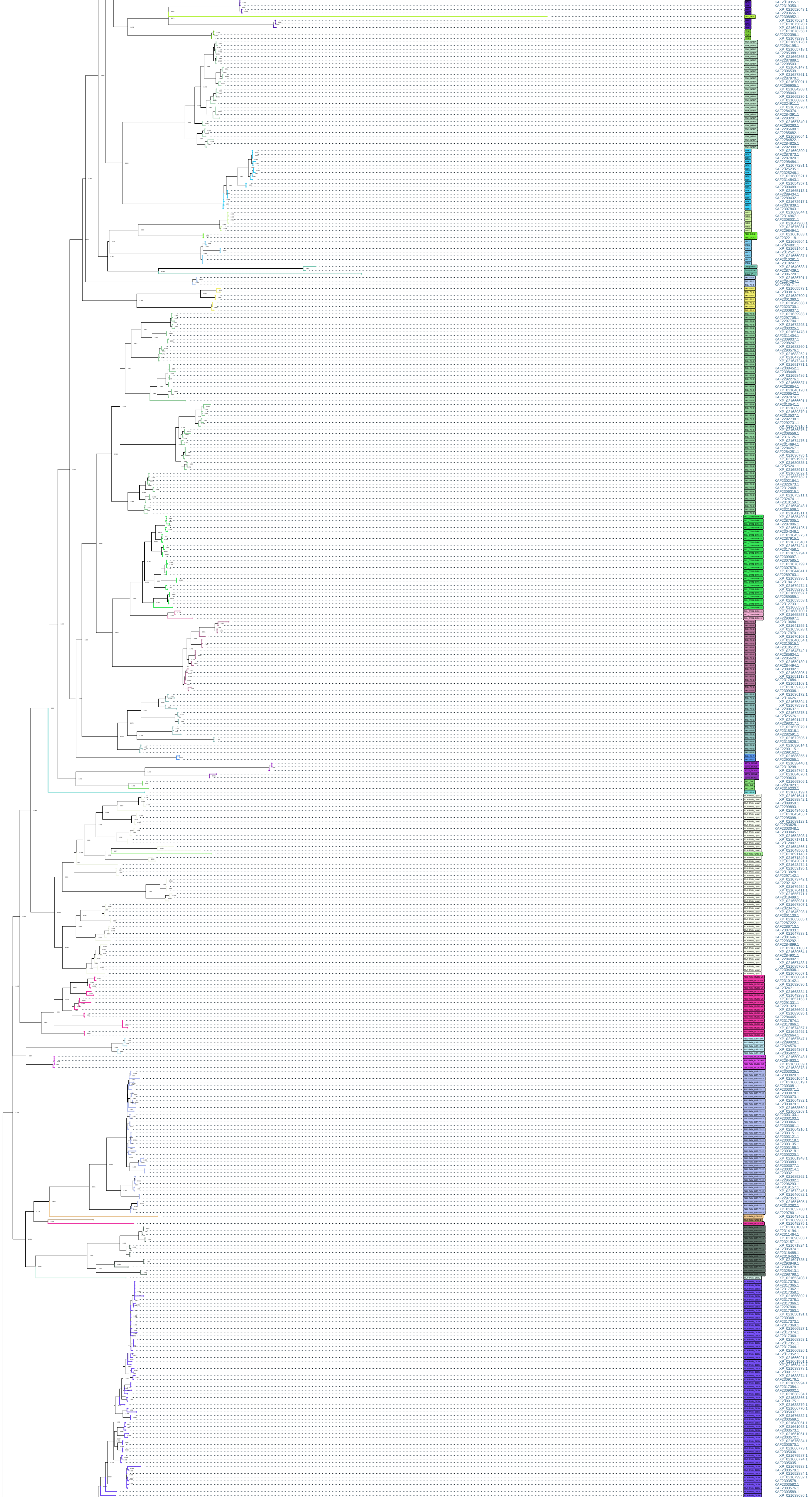

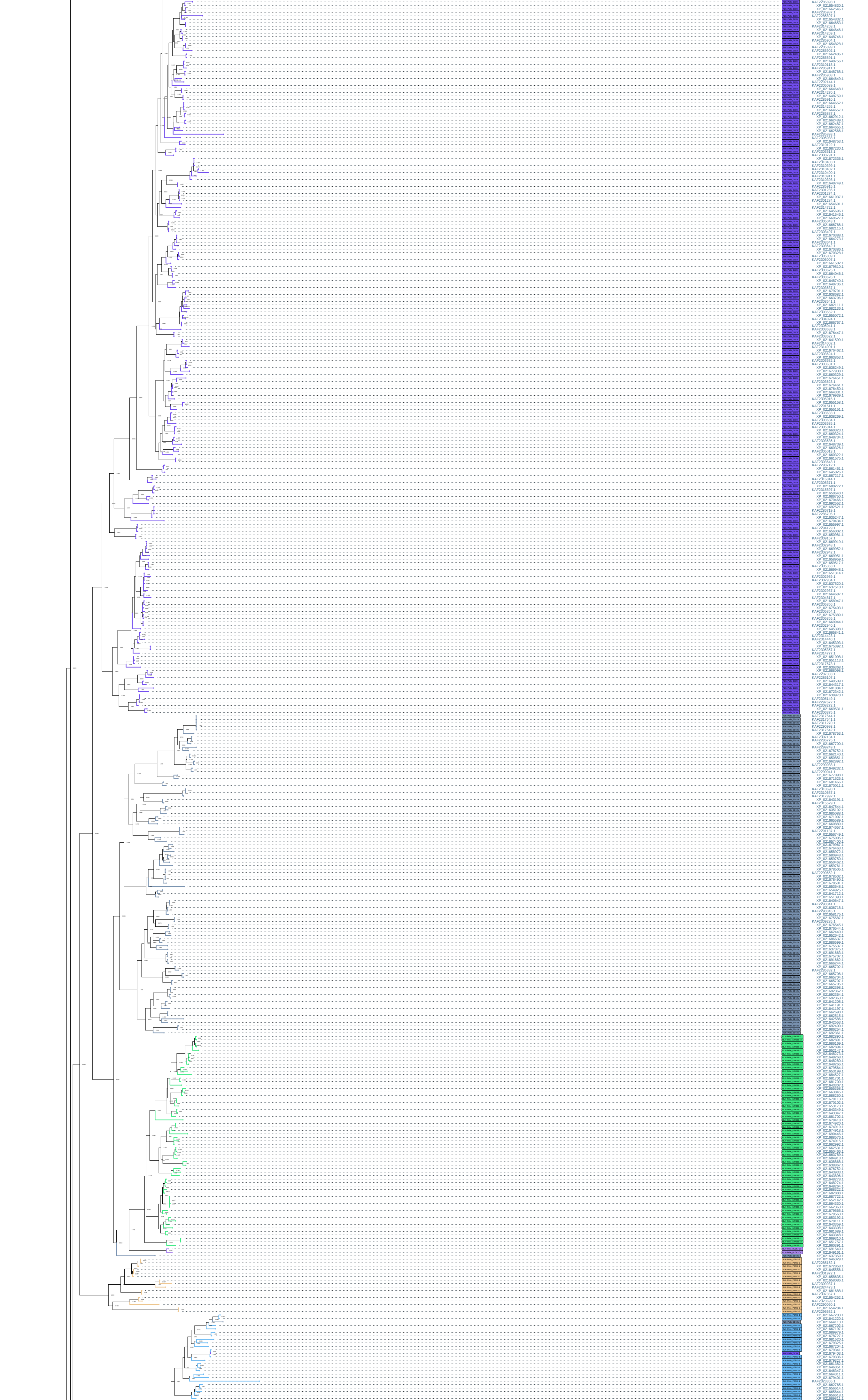

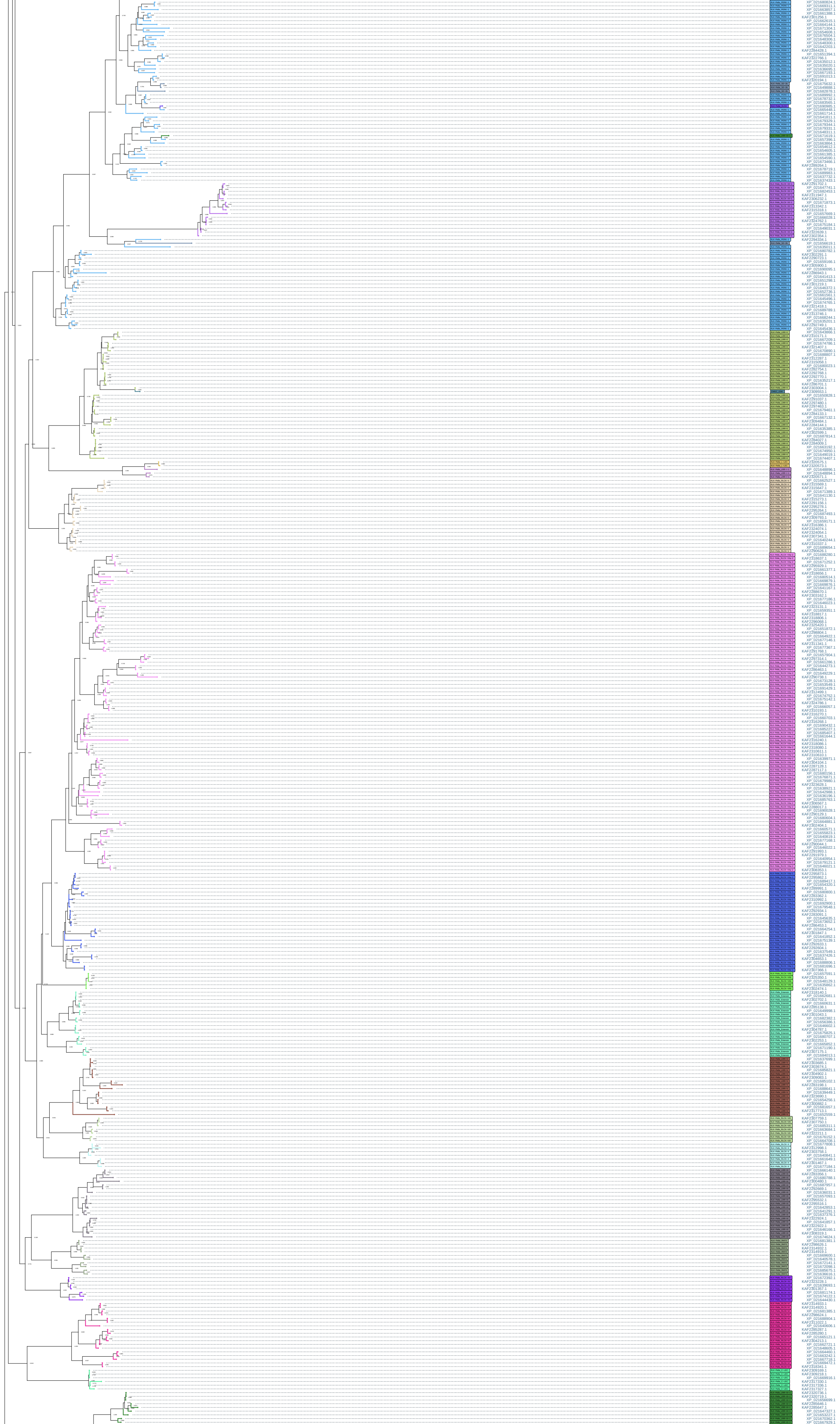

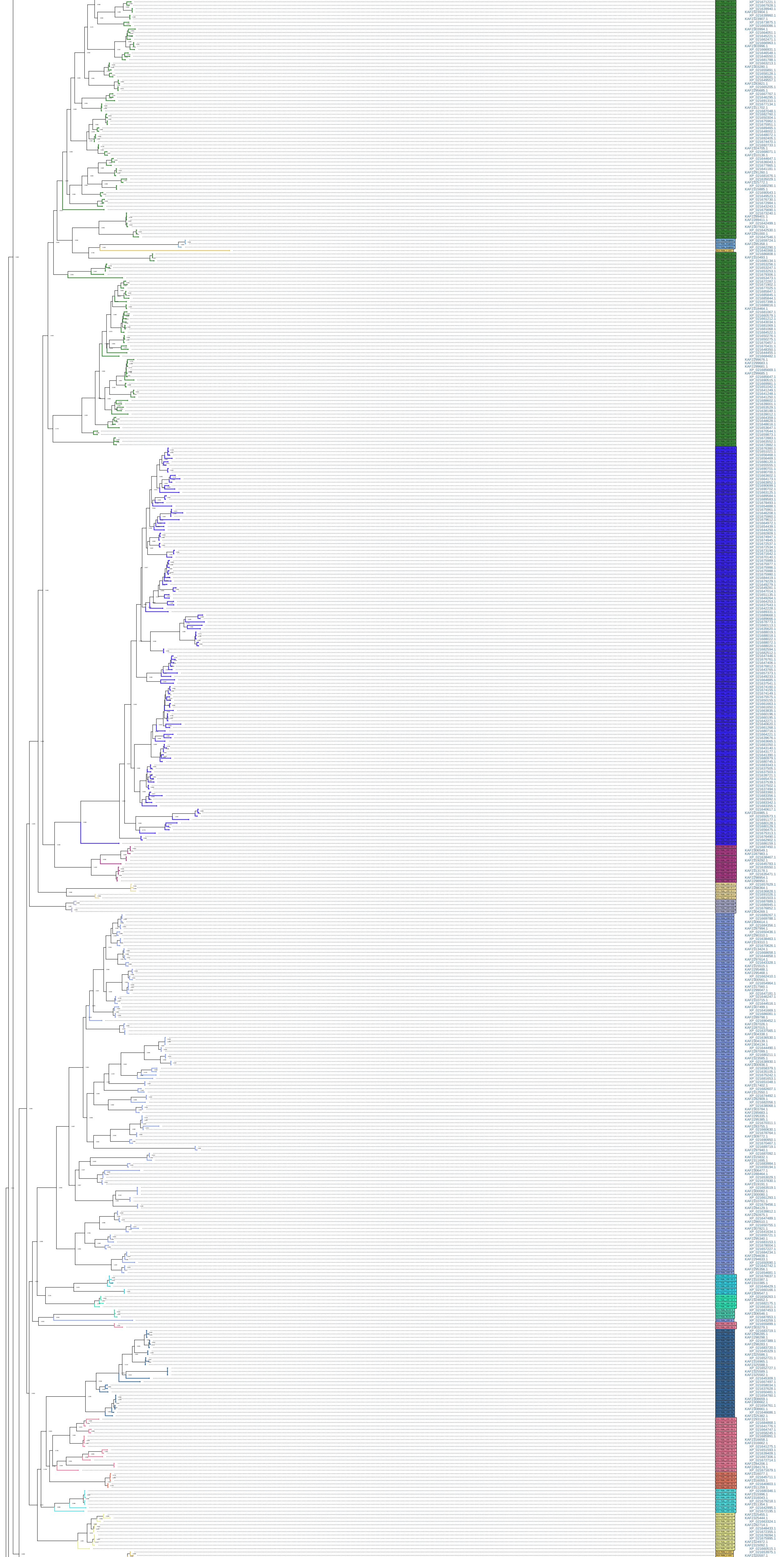

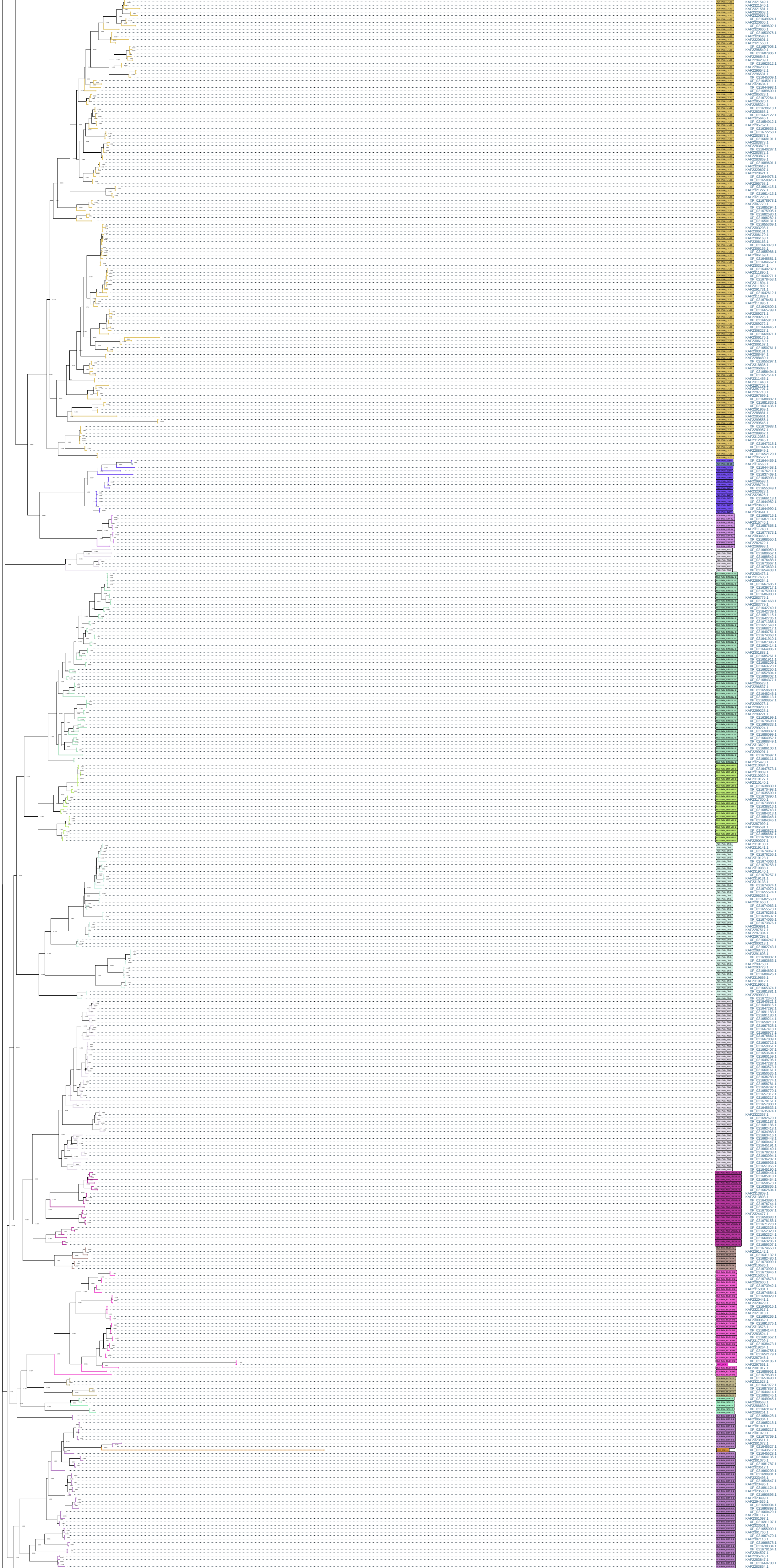

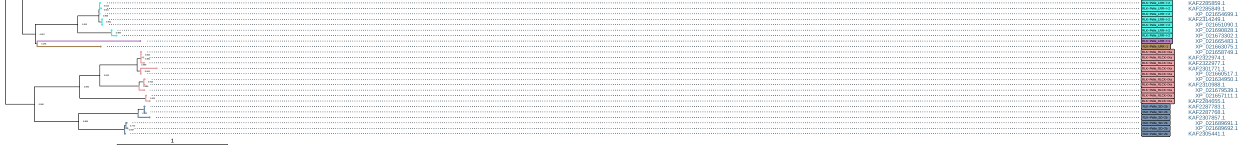
RLK-Pelle_LRR-I-2 KAF2285859.1
RLK-Pelle_LRR-I-2 KAF2285849.1
RLK-Pelle_LRR-I-2 XP_021654699.1
RLK-Pelle_LRR-I-2 KAF2314249.1
RLK-Pelle_LRR-I-2 XP_021651090.1
RLK-Pelle_LRR-I-2 XP_021690828.1
RLK-Pelle_LRR-I-2 XP_021673302.1
RLK-Pelle_LRR-I-1 XP_021665483.1
RLK-Pelle_URK-1 XP_021663075.1
RLK-Pelle_RLCK-IXa XP_021658749.1
RLK-Pelle_RLCK-IXa KAF2322974.1
RLK-Pelle_RLCK-IXa KAF2322977.1
RLK-Pelle_RLCK-IXa KAF2301771.1
RLK-Pelle_RLCK-IXa XP_021660517.1
RLK-Pelle_RLCK-IXa XP_021634950.1
RLK-Pelle_RLCK-IXa KAF2310988.1
RLK-Pelle_RLCK-IXa XP_021679539.1
RLK-Pelle_RLCK-IXa XP_021657111.1
RLK-Pelle_RLCK-IXa KAF2284655.1
RLK-Pelle_SD-2b KAF2287783.1
RLK-Pelle_SD-2b KAF2287768.1
RLK-Pelle_SD-2b KAF2307857.1
RLK-Pelle_SD-2b XP_021689691.1
RLK-Pelle_SD-2b XP_021689692.1
RLK-Pelle_SD-2b KAF2305441.1
1
