# Supplementary material for: The rubber tree kinome: Genome-wide characterization and insights into coexpression patterns associated with abiotic stress responses
Source: Front Plant Sci. 2023 Feb 7;14:1068202. doi: 10.3389/fpls.2023.1068202 (PMC9941580; doi:10.3389/fpls.2023.1068202)

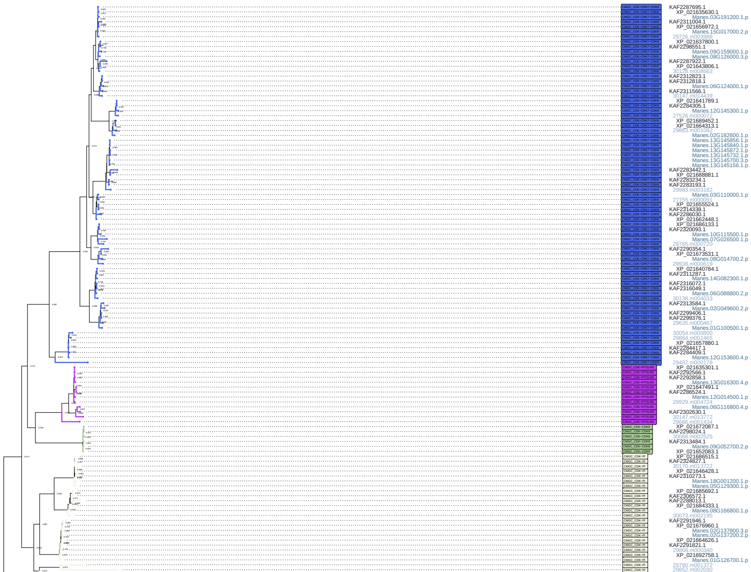
CDK-CRK7-CDK9
CDK-PITSLRE
CDK-CDK8
CDK-PI
KAF2287695.1
XP_021635630.1
Manes.03G191200.1.p
KAF2311004.1
XP_021656972.1
Manes.15G017000.2.p
29726.m003988
XP_021637800.1
KAF2298551.1
Manes.09G159000.1.p
Manes.08G126000.3.p
KAF2287922.1
XP_021643806.1
30128.m008663
KAF2312823.1
KAF2312818.1
Manes.06G124000.1.p
KAF2311566.1
30147.m014439
XP_021641789.1
KAF2284305.1
Manes.12G145300.1.p
27526.m000072
XP_021689452.1
XP_021664313.1
29863.m001082
Manes.02G182600.1.p
Manes.13G145856.1.p
Manes.13G145840.1.p
Manes.13G145872.1.p
Manes.13G145732.1.p
Manes.13G145700.3.p
Manes.13G145156.1.p
KAF2283442.1
XP_021688881.1
KAF2283234.1
KAF2283193.1
29983.m003182
Manes.03G110000.1.p
27755.m000091
XP_021655524.1
KAF2314338.1
KAF2286030.1
XP_021662448.1
XP_021686133.1
KAF2320093.1
Manes.10G115500.1.p
Manes.07G026500.1.p
29765.m000720
KAF2290354.1
XP_021673531.1
Manes.08G014700.2.p
29938.m000619
XP_021640784.1
KAF2311287.1
Manes.14G082300.1.p
KAF2316072.1
KAF2316049.1
Manes.06G088800.2.p
30138.m004033
KAF2313584.1
Manes.02G049600.2.p
KAF2299406.1
KAF2299376.1
29635.m000467
Manes.01G100500.1.p
30054.m000800
29864.m001465
XP_021657880.1
KAF2284417.1
KAF2284409.1
Manes.12G153600.4.p
29482.m000178
XP_021635301.1
KAF2292566.1
KAF2292858.1
Manes.13G016300.4.p
XP_021647491.1
KAF2286524.1
Manes.12G014500.1.p
29929.m004724
Manes.06G116800.4.p
KAF2302630.1
30147.m013772
29666.m001434
XP_021672087.1
KAF2298024.1
30068.m002525
KAF2313484.1
Manes.09G052700.2.p
XP_021652083.1
XP_021686515.1
KAF2324827.1
30170.m013722
XP_021646428.1
KAF2310273.1
Manes.18G001200.1.p
Manes.05G129300.1.p
XP_021685692.1
KAF2306572.1
KAF2288013.1
XP_021684333.1
Manes.08G166800.1.p
30073.m002195
KAF2291946.1
XP_021676960.1
Manes.02G137800.3.p
Manes.02G137200.2.p
XP_021664626.1
KAF2291821.1
29868.m000340
XP_021692758.1
Manes.01G126700.1.p
29780.m001372
29852.m002030

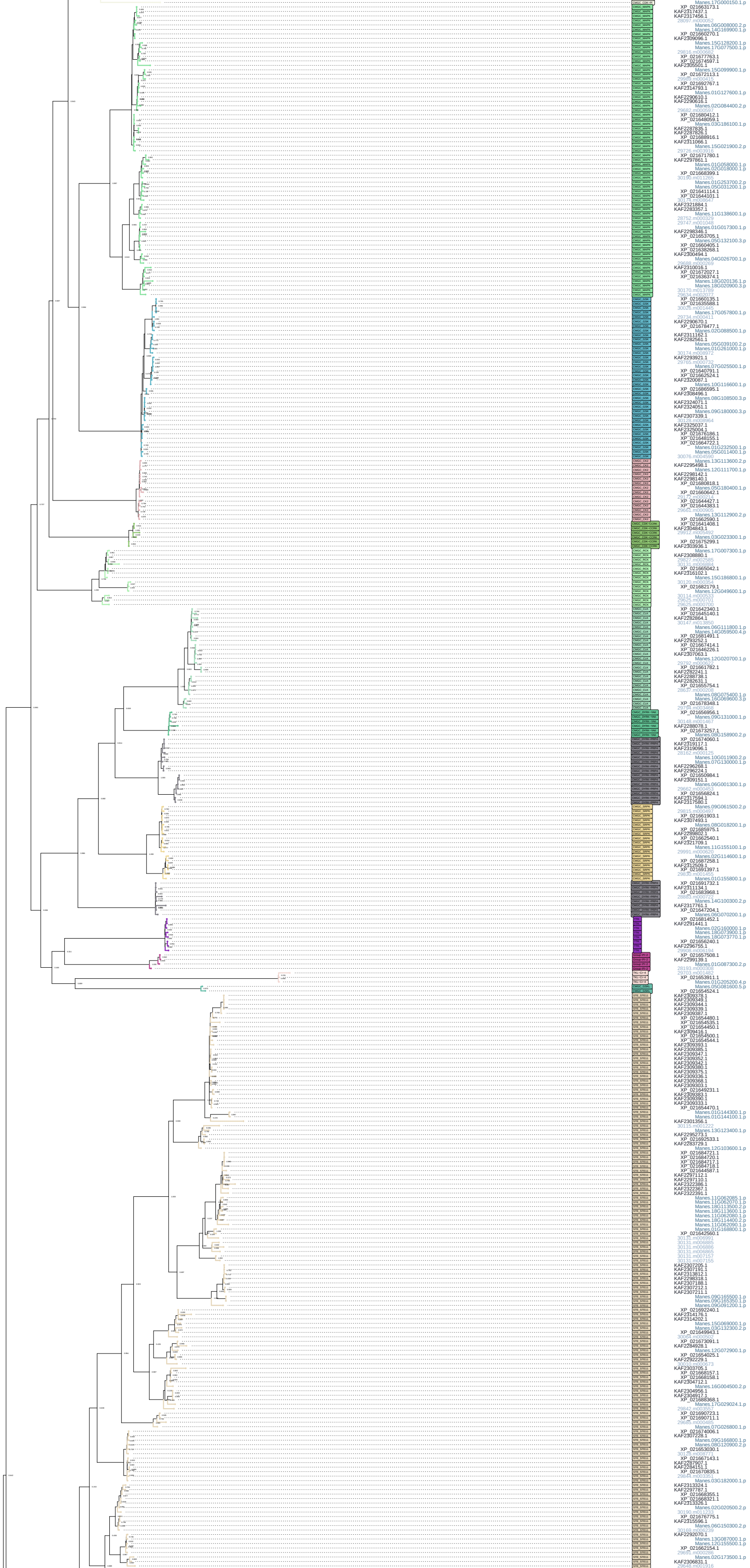

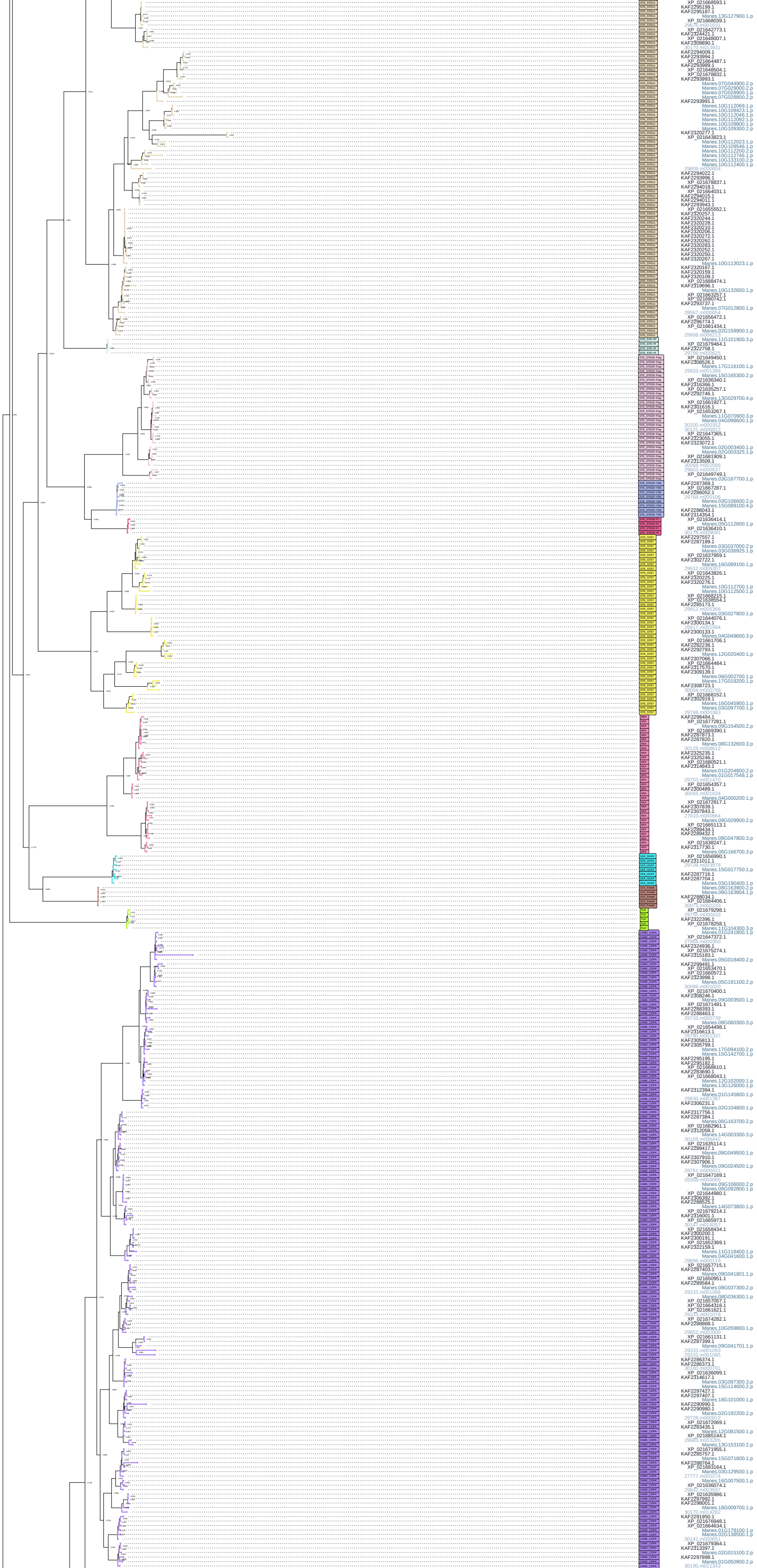

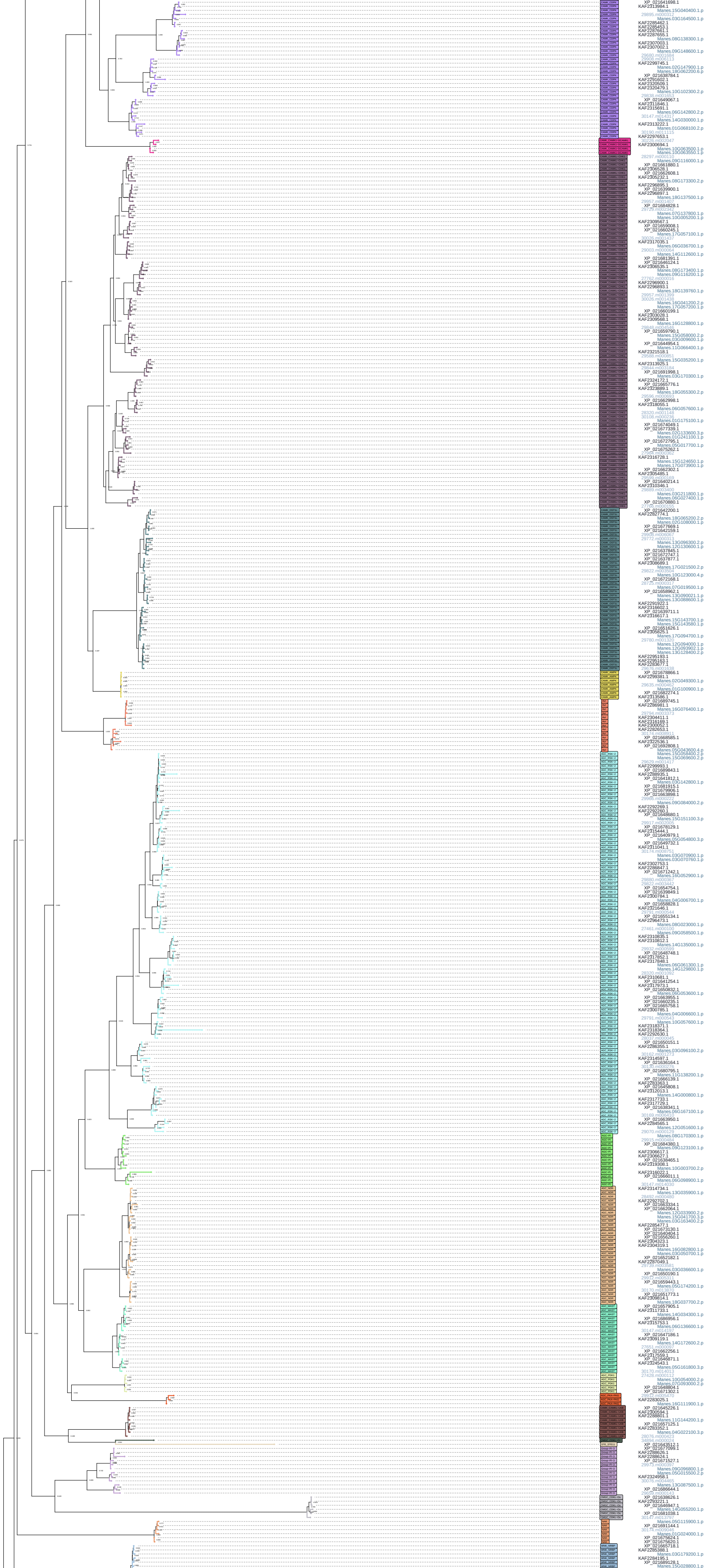

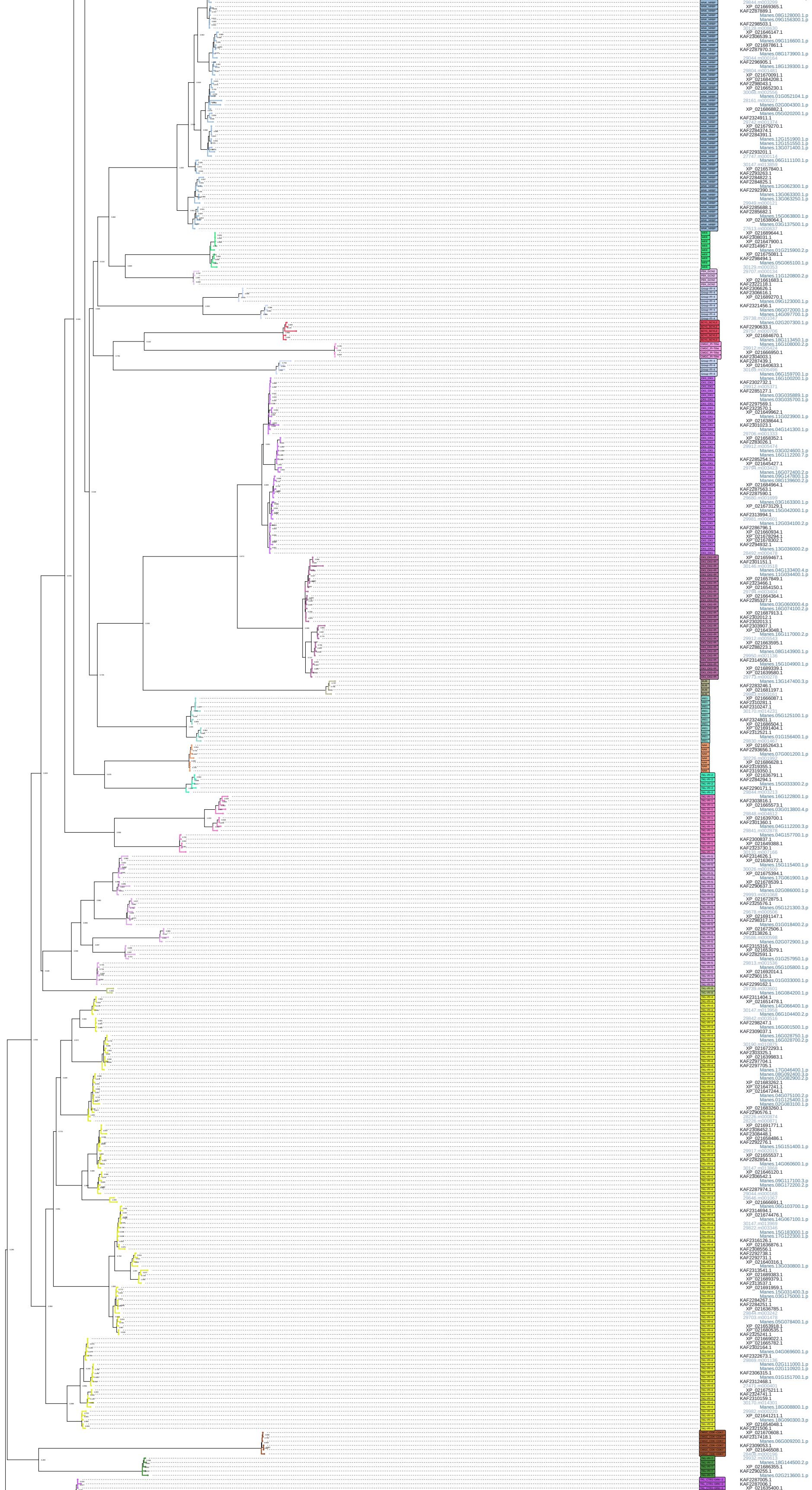

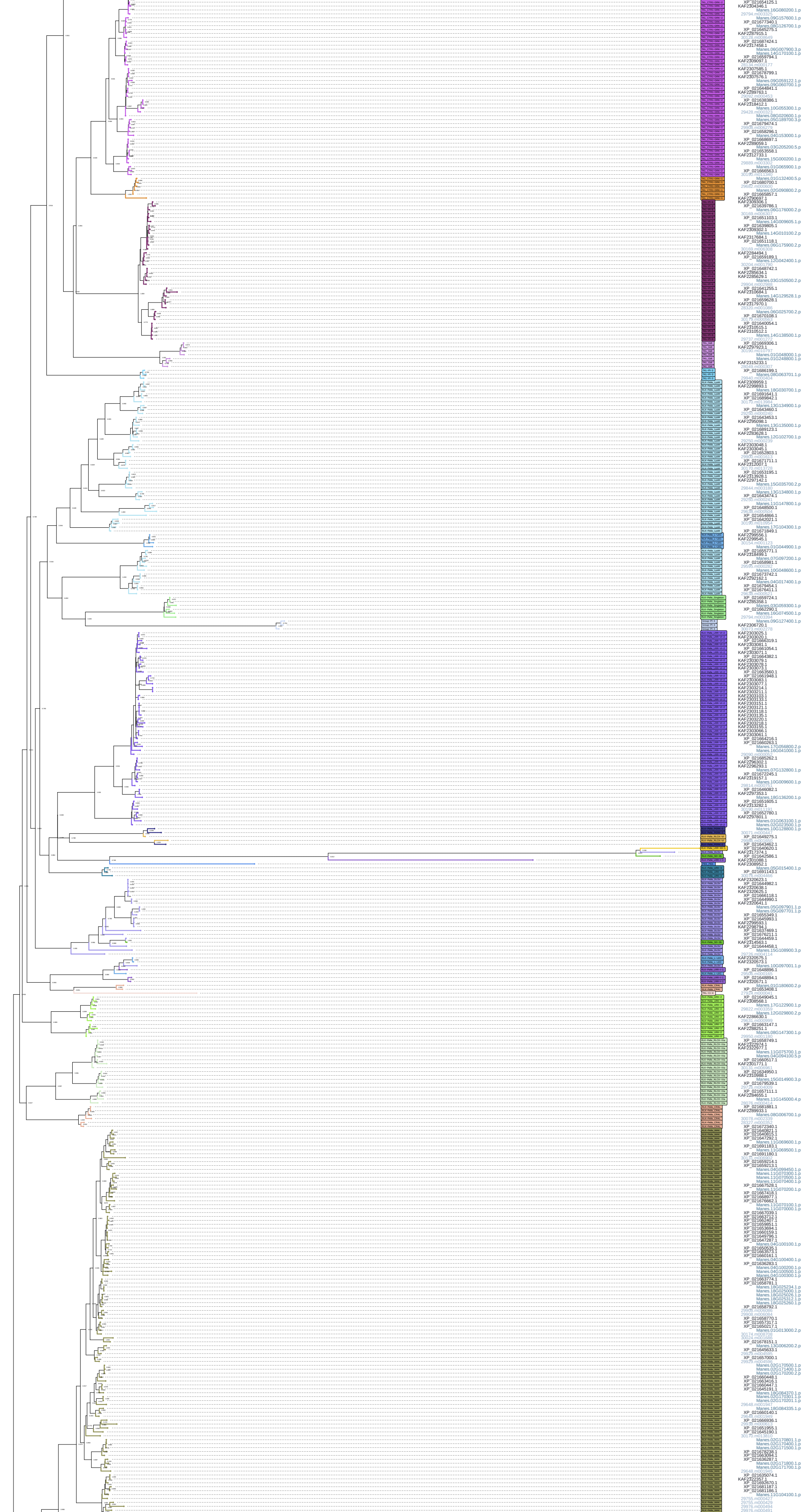

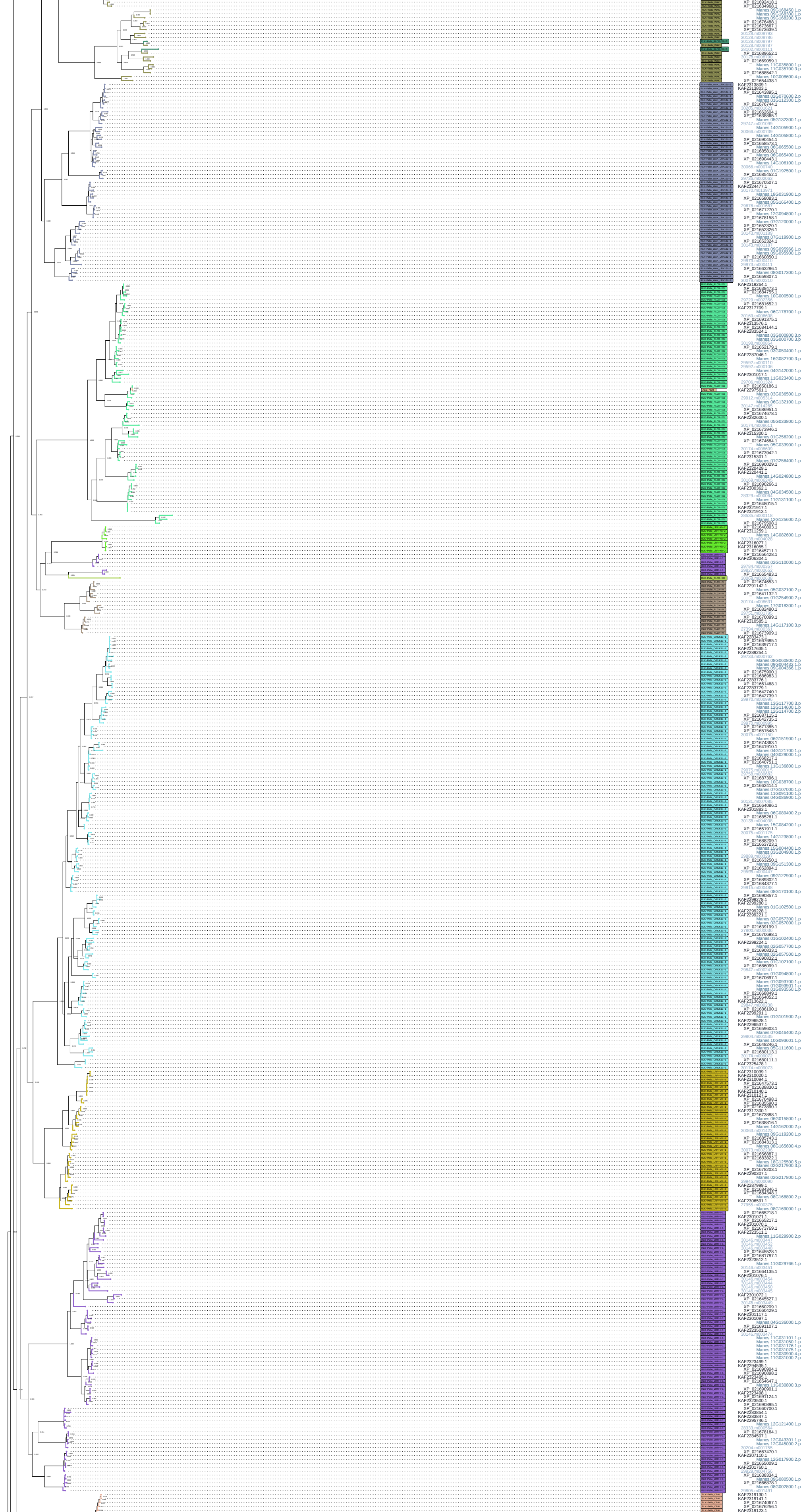

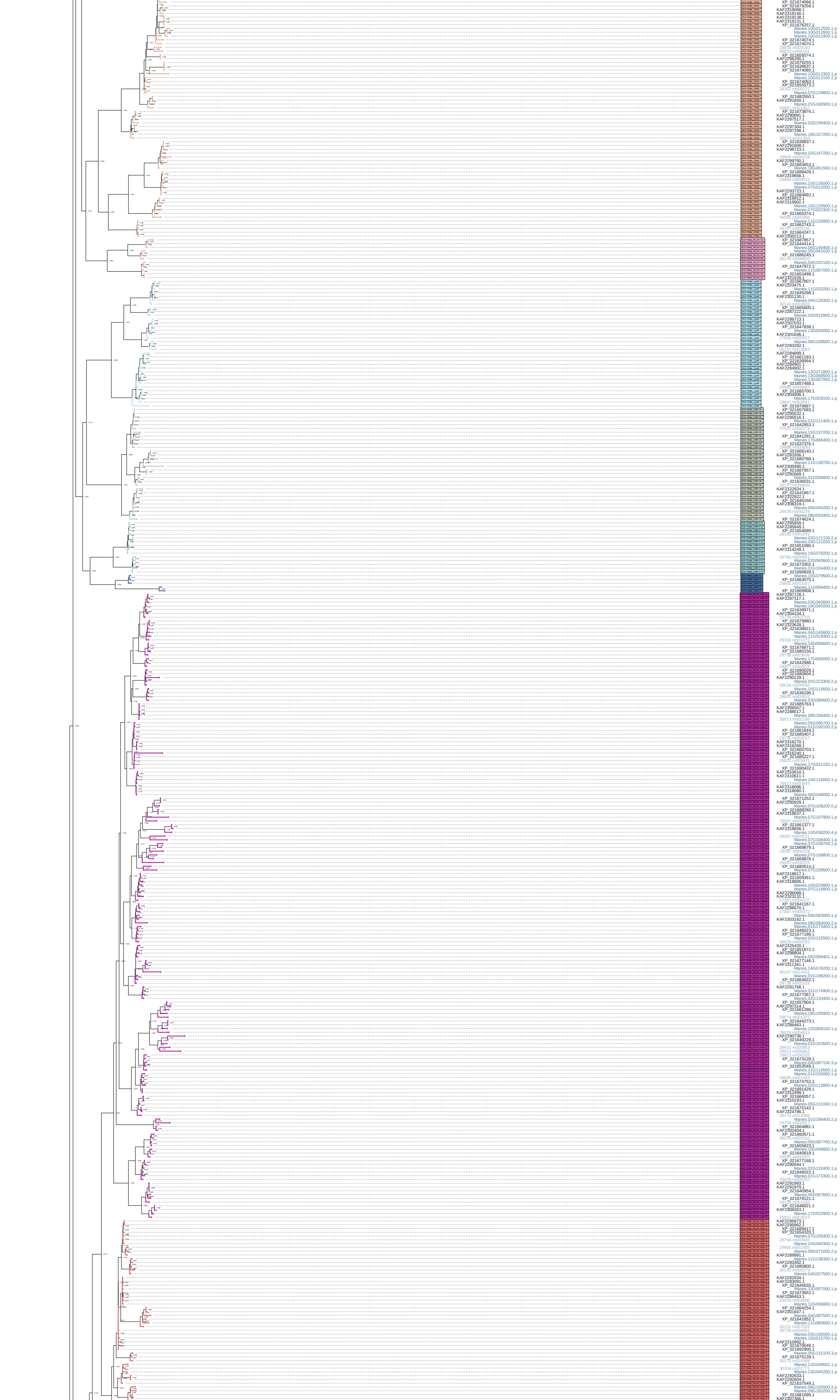

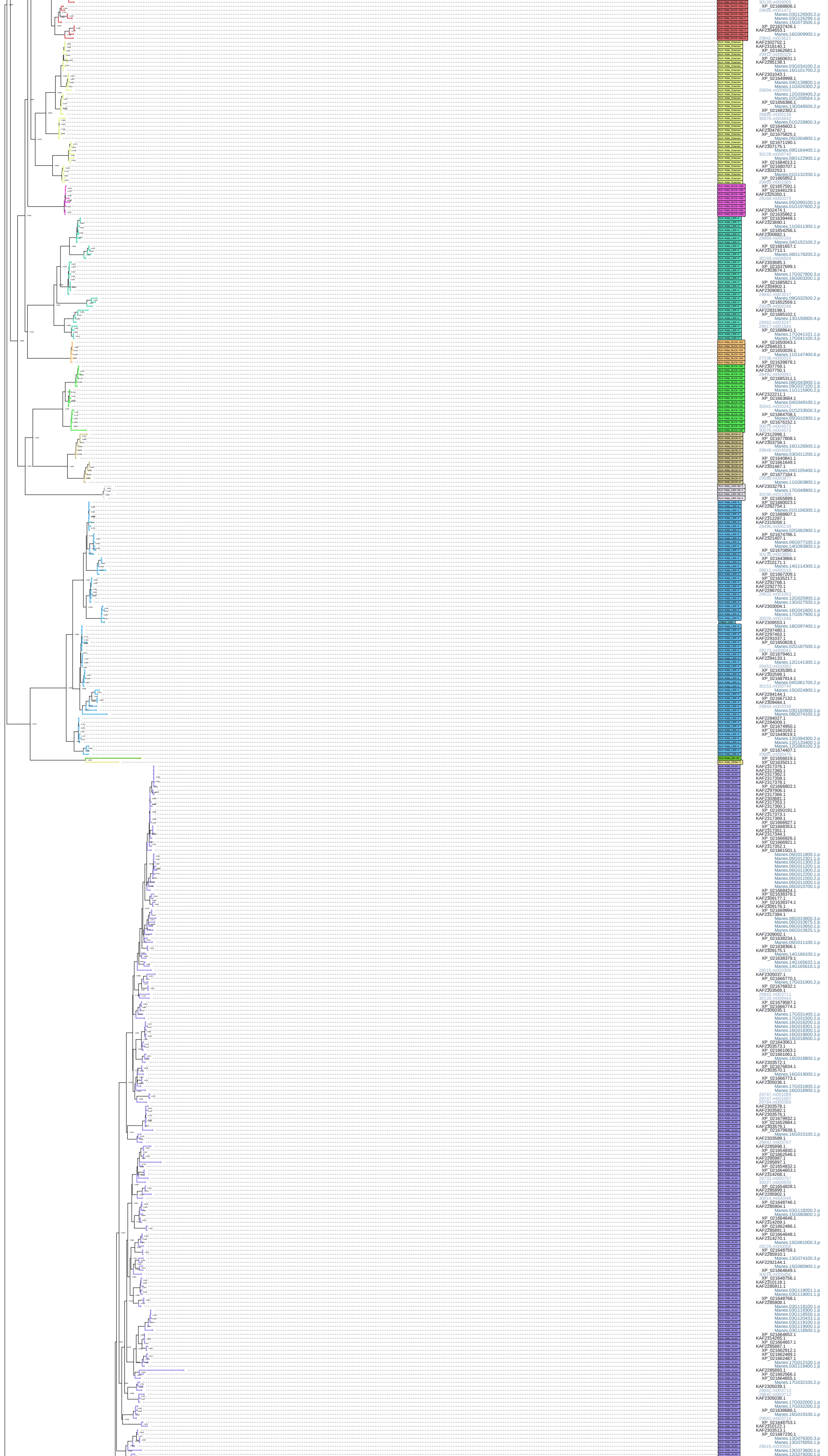

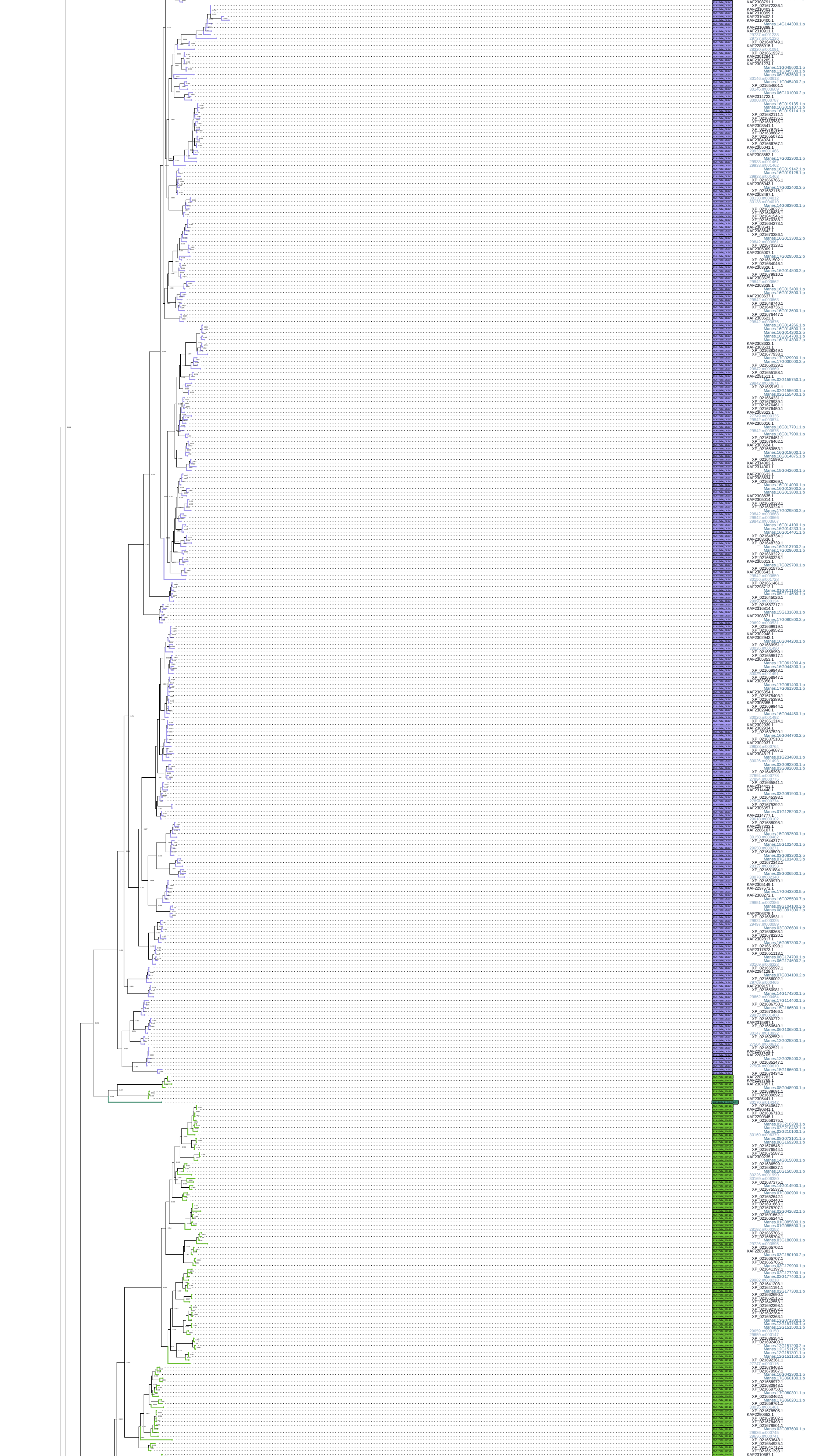
DLSV
KAF2308791.1
XP_021672336.1
KAF2310403.1
KAF2310399.1
KAF2310402.1
KAF2310400.1
Manes.14G144300.1.p
KAF2310398.1
KAF2310911.1
29737.m001238
29737.m001236
XP_021648749.1
KAF2285915.1
28320.m001091
XP_021661937.1
KAF2301284.1
KAF2301285.1
KAF2301274.1
Manes.11G045600.1.p
Manes.11G045500.1.p
Manes.06G053500.1.p
30146.m003613
Manes.11G045400.2.p
XP_021654601.1
30146.m003609
Manes.06G101000.2.p
KAF2314722.1
30008.m000787
Manes.16G019135.1.p
Manes.16G019107.1.p
Manes.16G019114.1.p
XP_021682111.1
XP_021682136.1
XP_021663796.1
KAF2303541.1
XP_021679791.1
XP_021638682.1
XP_021655072.1
KAF2304024.1
XP_021666767.1
KAF2305041.1
29933.m001466
KAF2303552.1
Manes.17G032300.1.p
29933.m001467
29933.m001462
Manes.16G019142.1.p
Manes.16G019128.1.p
29933.m001463
XP_021666766.1
KAF2305043.1
Manes.17G032400.3.p
XP_021682115.1
KAF2303497.1
30138.m004012
30138.m004010
Manes.14G083900.1.p
XP_021669627.1
XP_021645696.1
XP_021641546.1
XP_021670388.1
XP_021664273.1
KAF2303641.1
KAF2303642.1
XP_021670386.1
Manes.16G013300.2.p
29842.m003661
XP_021670328.1
KAF2305009.1
KAF2305007.1
Manes.17G029500.2.p
XP_021661502.1
XP_021664046.1
KAF2303626.1
Manes.16G014800.2.p
XP_021679810.1
KAF2303625.1
29842.m003662
KAF2303638.1
Manes.16G013400.1.p
Manes.16G013500.1.p
KAF2303637.1
29842.m003663
XP_021648740.1
XP_021648736.1
Manes.16G013600.1.p
XP_021676447.1
KAF2303622.1
29842.m003676
Manes.16G014266.1.p
Manes.16G014500.1.p
Manes.16G014200.2.p
Manes.16G014700.1.p
Manes.16G014300.2.p
KAF2303632.1
KAF2303631.1
XP_021638249.1
XP_021677938.1
Manes.17G029900.1.p
Manes.17G030000.2.p
XP_021660329.1
29842.m003669
XP_021655158.1
KAF2291511.1
Manes.02G155750.1.p
29842.m003671
XP_021655151.1
Manes.02G155600.1.p
Manes.02G155400.1.p
XP_021664331.1
XP_021679939.1
XP_021676461.1
XP_021676450.1
KAF2303623.1
27749.m000335
29842.m003674
KAF2305016.1
Manes.16G017701.1.p
29842.m003675
Manes.16G017900.1.p
XP_021676451.1
XP_021676462.1
KAF2303624.1
XP_021663853.1
Manes.16G018000.1.p
Manes.16G014875.1.p
XP_021641599.1
KAF2314002.1
KAF2314001.1
Manes.15G042600.1.p
KAF2303633.1
KAF2303634.1
XP_021638269.1
Manes.16G014000.1.p
Manes.16G013900.2.p
Manes.16G013800.1.p
KAF2303635.1
KAF2305014.1
XP_021660323.1
XP_021660324.1
Manes.17G029800.2.p
29842.m003668
29842.m003666
29842.m003667
Manes.16G014100.1.p
Manes.16G014233.1.p
Manes.16G014401.1.p
XP_021648734.1
KAF2303636.1
XP_021648739.1
Manes.16G013700.2.p
Manes.17G029600.1.p
XP_021660322.1
XP_021660326.1
KAF2305013.1
Manes.17G029700.1.p
XP_021661575.1
KAF2303643.1
29842.m003659
30156.m001728
XP_021661461.1
KAF2298712.1
Manes.01G011184.1.p
Manes.05G114600.1.p
XP_021645026.1
29996.m000134
XP_021687217.1
KAF2316814.1
Manes.15G131600.1.p
KAF2308371.1
Manes.17G080800.2.p
29692.m000531
XP_021669919.1
XP_021669952.1
KAF2302948.1
KAF2302942.1
Manes.16G044200.1.p
XP_021669951.1
30026.m001490
XP_021658959.1
XP_021659517.1
KAF2305353.1
Manes.17G061200.4.p
Manes.16G044300.1.p
XP_021669948.1
30026.m001491
XP_021658947.1
KAF2305356.1
Manes.17G061400.1.p
Manes.17G061300.1.p
KAF2305354.1
XP_021675403.1
XP_021675389.1
KAF2305355.1
XP_021669944.1
KAF2302940.1
Manes.16G044450.1.p
30026.m001492
XP_021651314.1
KAF2302939.1
KAF2302934.1
XP_021637520.1
Manes.16G044700.2.p
XP_021637510.1
KAF2302937.1
29628.m000764
XP_021664687.1
KAF2304817.1
Manes.01G234800.1.p
30026.m001493
Manes.03G092300.1.p
Manes.03G092000.1.p
XP_021645398.1
27894.m000778
27894.m000775
XP_021665841.1
KAF2314423.1
KAF2314440.1
Manes.03G091900.1.p
XP_021645393.1
27894.m000774
XP_021675392.1
KAF2305357.1
Manes.01G125200.2.p
KAF2314777.1
29618.m000102
XP_021688098.1
KAF2287333.1
KAF2286107.1
Manes.15G092500.1.p
30150.m000482
XP_021644317.1
Manes.15G102400.1.p
29650.m000271
XP_021649509.1
Manes.03G083200.2.p
Manes.07G101400.3.p
XP_021672342.1
28327.m000353
XP_021681884.1
Manes.08G006500.1.p
30078.m002340
XP_021639970.1
KAF2305149.1
KAF2297672.1
Manes.17G043300.5.p
KAF2308272.1
Manes.16G025500.7.p
29851.m002386
Manes.09G104100.2.p
Manes.08G091300.2.p
KAF2306375.1
XP_021669531.1
29624.m000325
29497.m000089
Manes.03G076600.1.p
XP_021636368.1
XP_021678220.1
KAF2302817.1
Manes.16G057300.2.p
XP_021651098.1
KAF2317673.1
XP_021651113.1
Manes.06G174700.1.p
Manes.06G174600.2.p
30169.m006328
XP_021655997.1
KAF2294129.1
Manes.07G034100.2.p
XP_021656002.1
29769.m000465
KAF2309157.1
XP_021650981.1
Manes.14G174200.1.p
29662.m000464
Manes.17G114400.1.p
XP_021686750.1
Manes.15G166500.1.p
XP_021670466.1
29933.m001408
XP_021680272.1
KAF2315897.1
XP_021650640.1
Manes.06G106800.1.p
30147.m013922
XP_021692552.1
Manes.12G025300.1.p
27504.m000612
XP_021692521.1
KAF2286719.1
KAF2286705.1
Manes.12G025400.2.p
XP_021635247.1
27504.m000610
Manes.15G166600.1.p
XP_021670434.1
SD-2b
KAF2287783.1
KAF2287768.1
KAF2307857.1
Manes.08G048900.1.p
XP_021689691.1
XP_021689692.1
KAF2305441.1
RLCK-XII-2
30170.m014242
XP_021640647.1
KAF2290341.1
XP_021636718.1
KAF2290345.1
XP_021658175.1
Manes.02G210200.1.p
Manes.02G210432.1.p
Manes.02G210100.1.p
30169.m006379
Manes.08G073101.1.p
Manes.06G169200.1.p
XP_021676545.1
XP_021676544.1
XP_021675587.1
KAF2309235.1
Manes.14G015000.1.p
XP_021686599.1
XP_021686637.1
Manes.10G150500.1.p
30226.m001990
30169.m006380
XP_021637375.1
Manes.14G014900.1.p
XP_021675537.1
Manes.07G000900.1.p
XP_021652642.1
XP_021662440.1
XP_021691663.1
XP_021675707.1
Manes.02G042632.1.p
XP_021691662.1
XP_021666244.1
Manes.01G085600.1.p
Manes.01G085500.1.p
28192.m000252
XP_021665706.1
XP_021665704.1
Manes.03G180000.1.p
29726.m003895
XP_021665702.1
KAF2285382.1
Manes.03G180100.2.p
XP_021665707.1
XP_021665705.1
Manes.03G179900.1.p
XP_021641197.1
Manes.02G177200.1.p
Manes.02G177400.1.p
29982.m000218
XP_021641208.1
XP_021641191.1
Manes.02G177300.1.p
XP_021662690.1
XP_021662515.1
XP_021642553.1
XP_021692398.1
XP_021692362.1
XP_021692364.1
XP_021692363.1
Manes.13G071300.1.p
Manes.12G151750.1.p
Manes.12G151500.1.p
29659.m000150
29659.m000147
XP_021686254.1
XP_021692400.1
Manes.12G151200.2.p
Manes.12G151125.1.p
Manes.12G151301.1.p
Manes.12G151150.1.p
XP_021692361.1
27747.m000116
XP_021676463.1
XP_021679967.1
Manes.16G042300.1.p
Manes.17G060100.1.p
XP_021658972.1
XP_021680948.1
XP_021659750.1
Manes.17G060301.1.p
XP_021650462.1
Manes.17G060201.1.p
XP_021659761.1
30026.m001481
XP_021678505.1
KAF2290652.1
XP_021678502.1
XP_021678490.1
XP_021678501.1
Manes.02G087600.1.p
29636.m000745
29636.m000741
XP_021653648.1
XP_021654925.1
XP_021641712.1
XP_021651393.1
KAF2310687.1

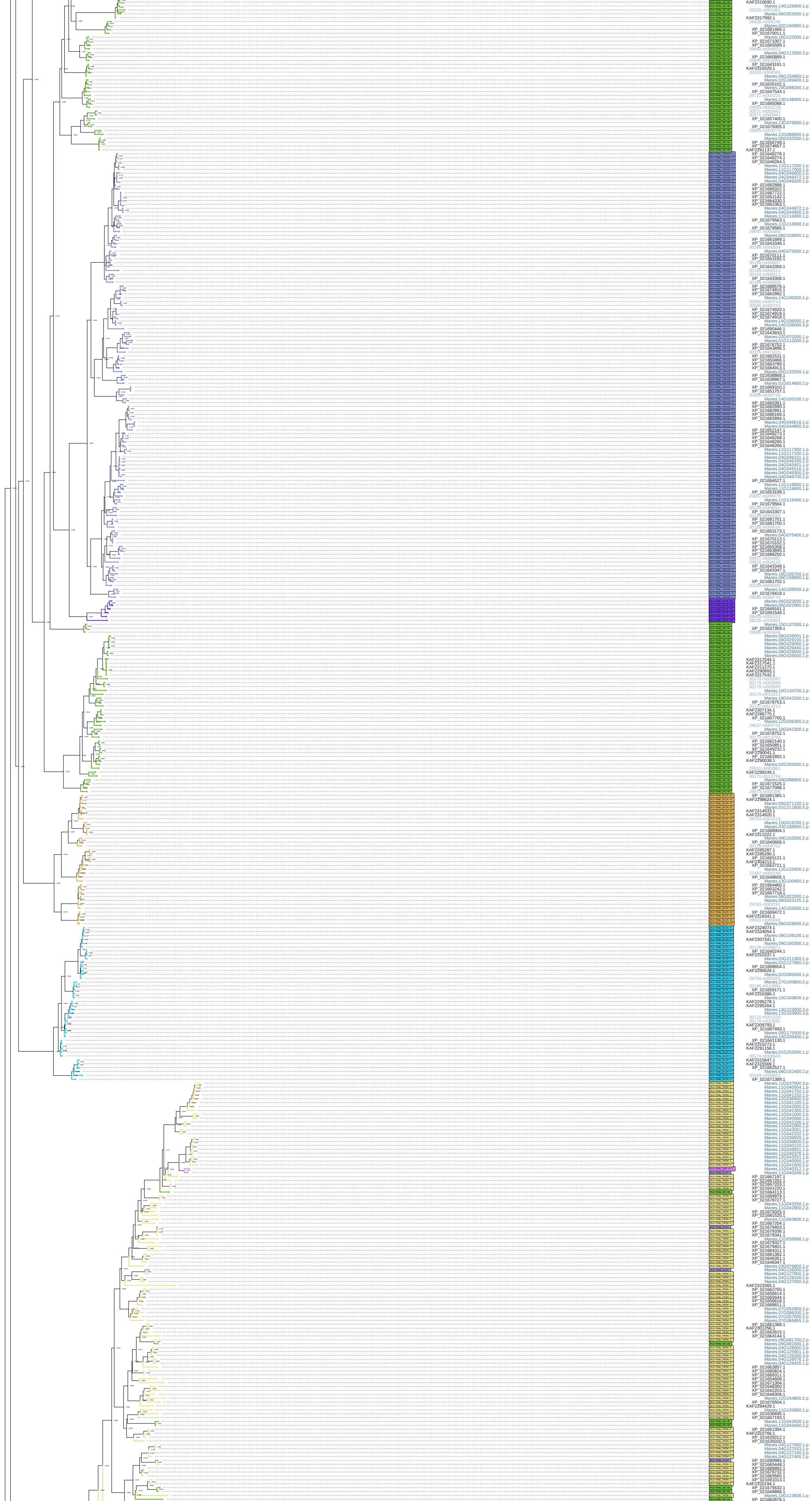

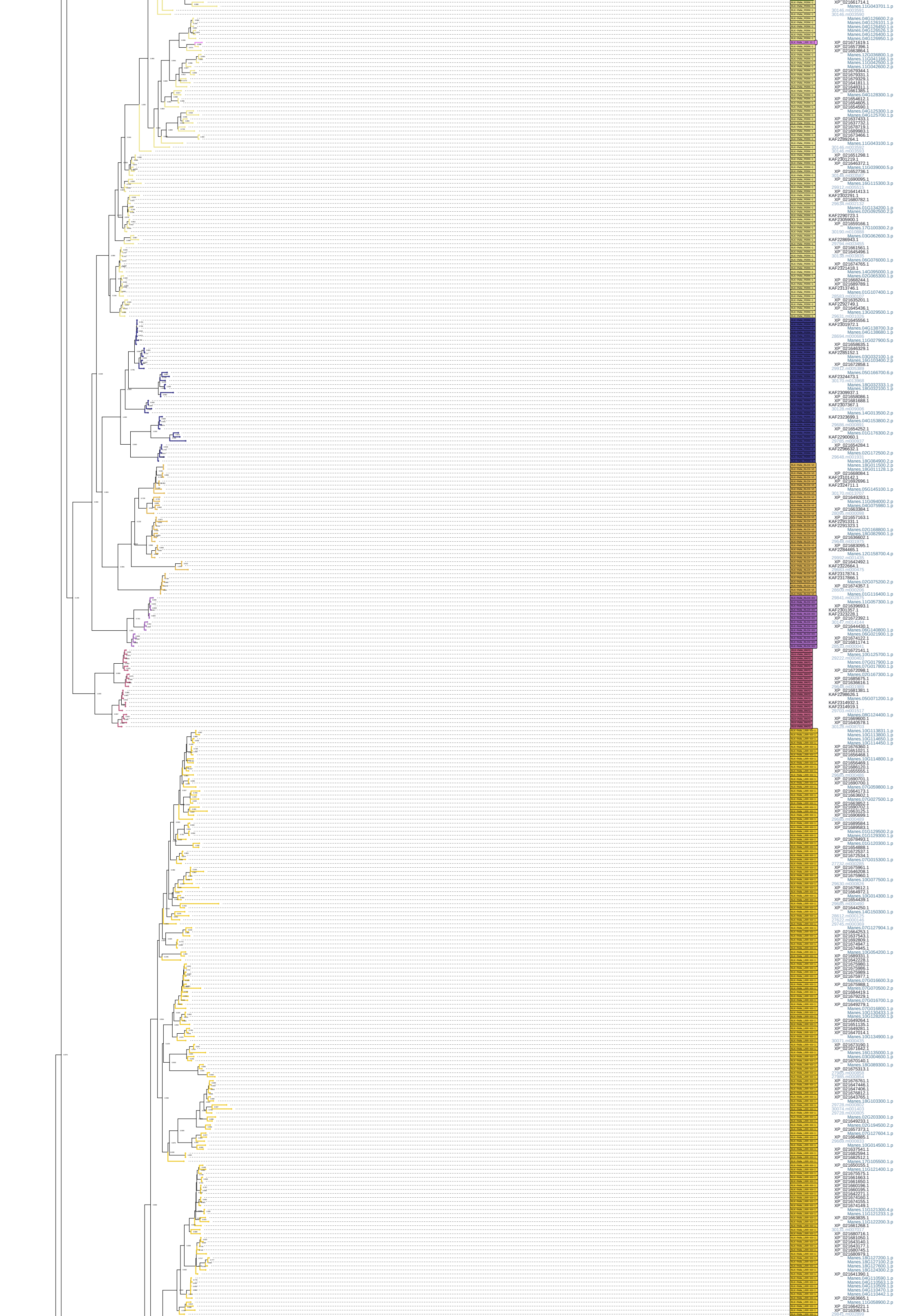

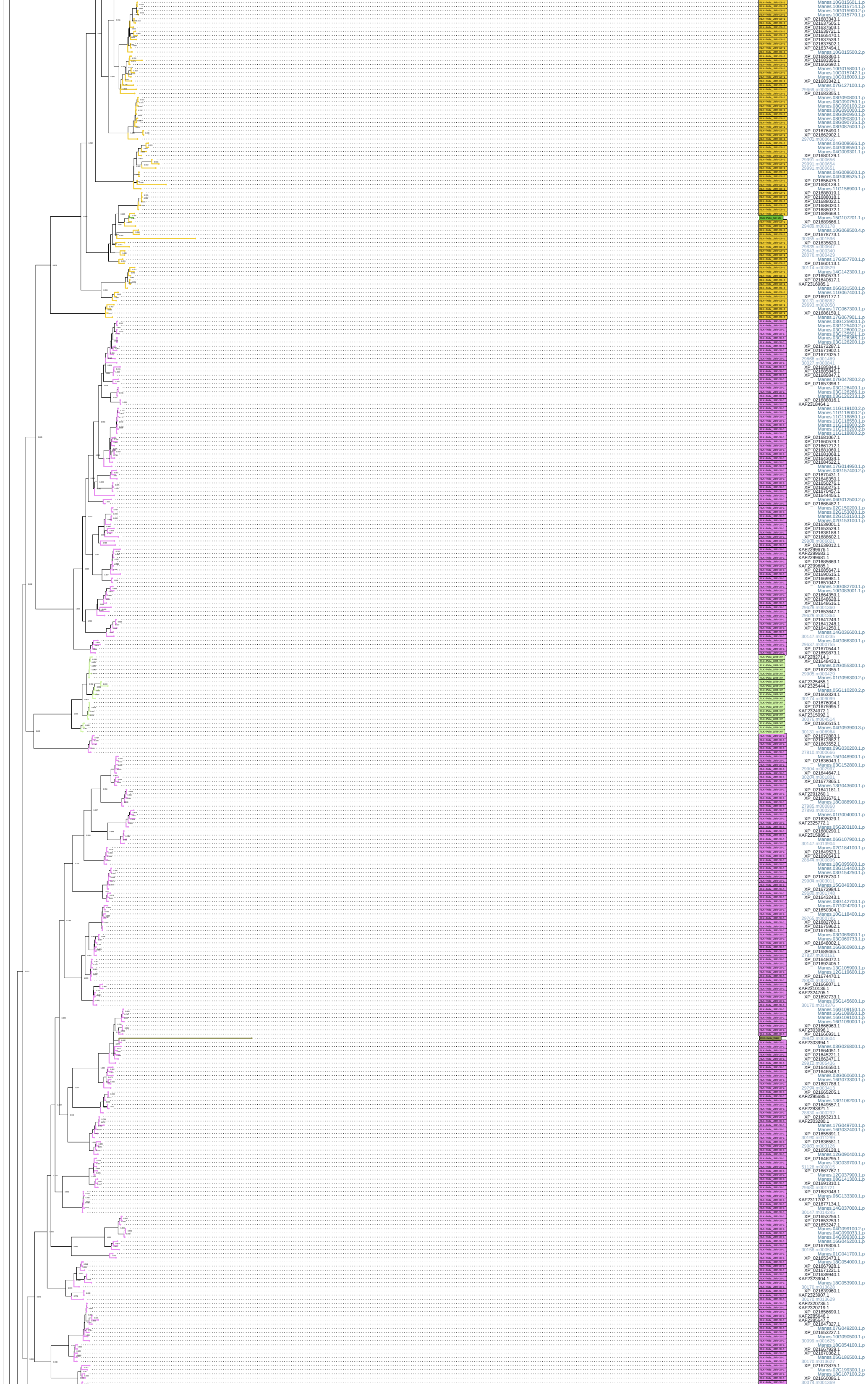

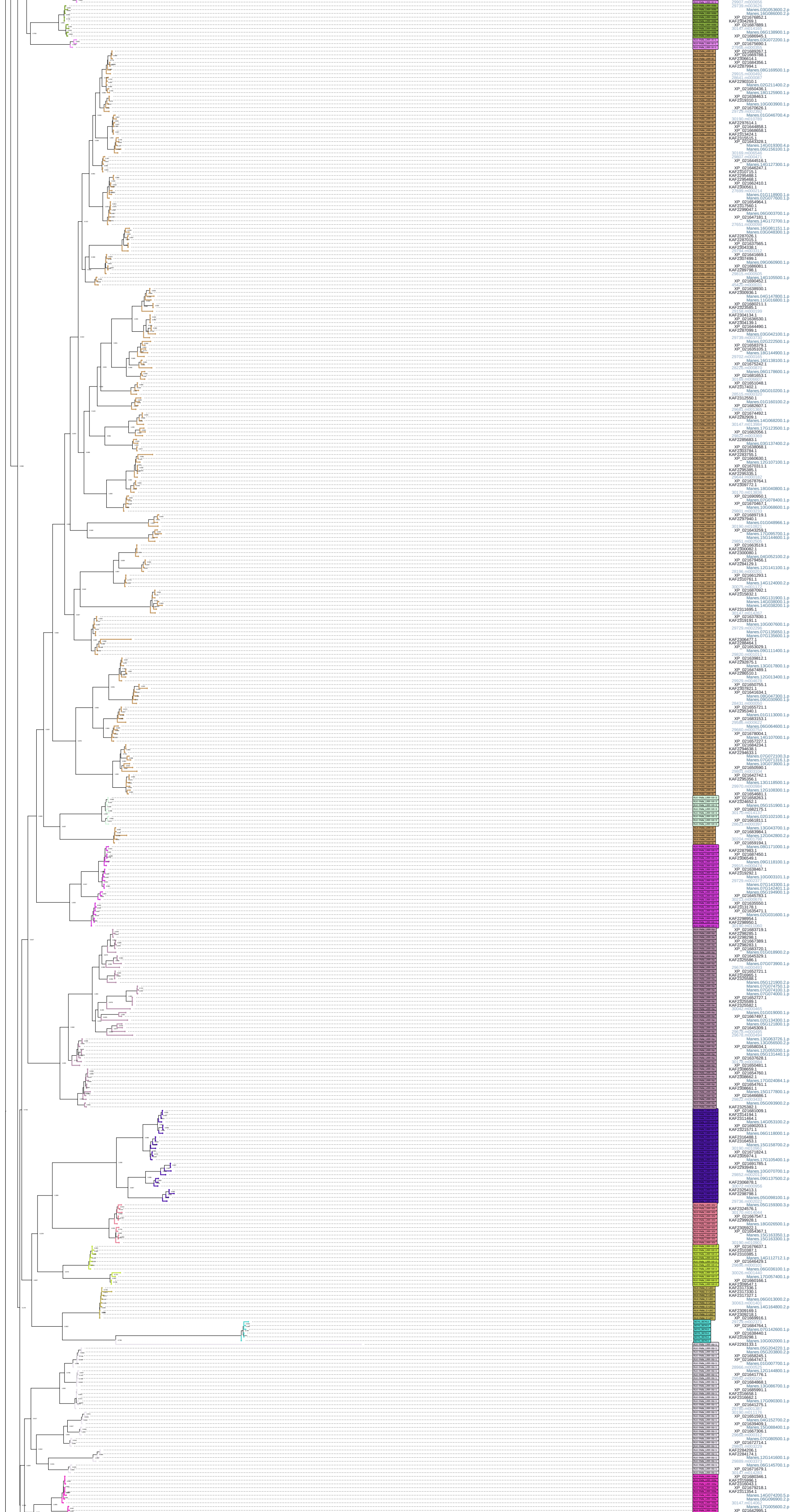

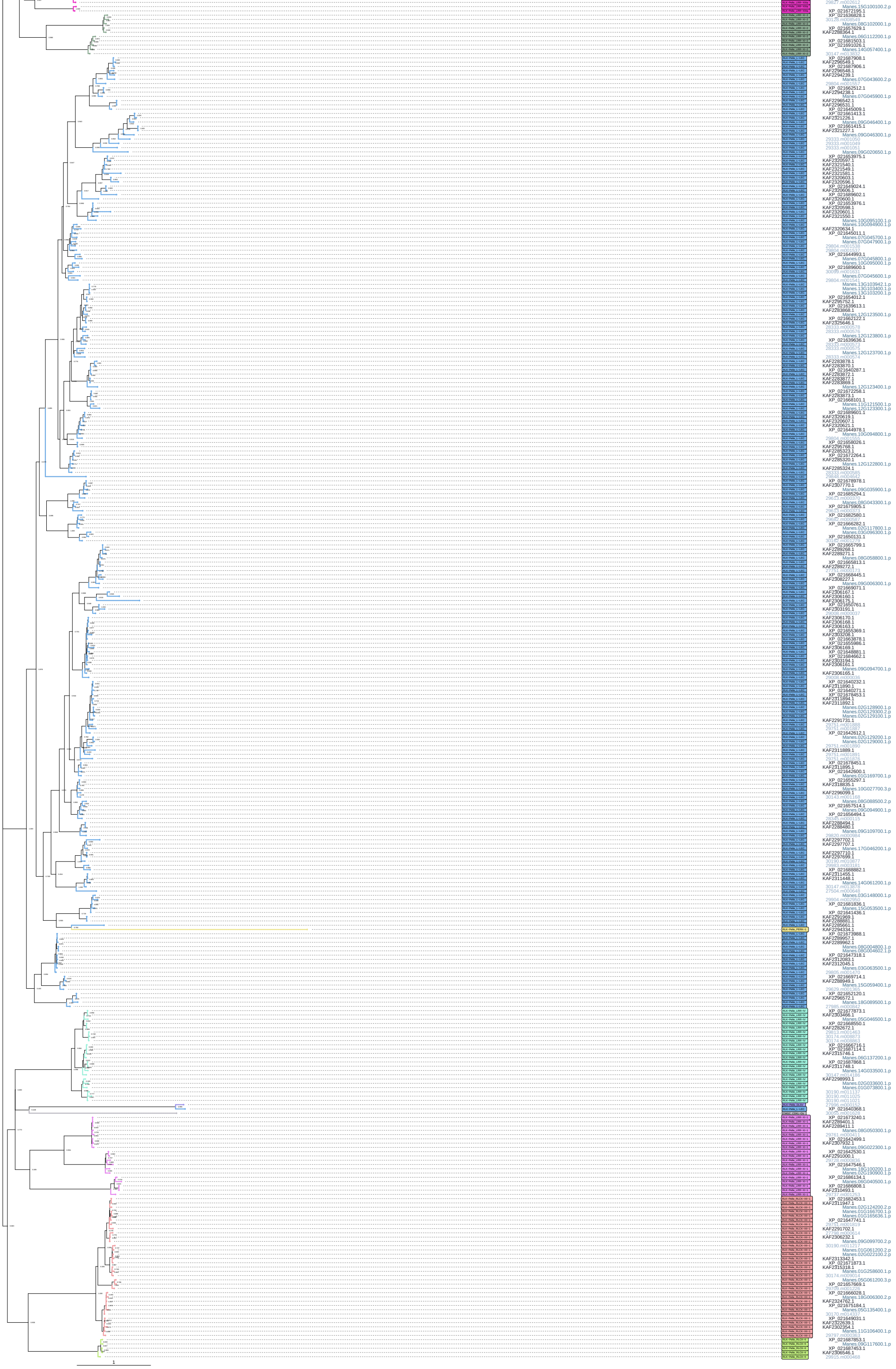

Supplement: Supplementary file 2 [file Image_2.pdf]
